# Decoding Heat-Shock Responses in *Vitis vinifera* L. by Metabolic, VOC, and Physiological Profiling: Toward the Identification of Volatile and Metabolic Biomarkers

**DOI:** 10.1021/acs.jafc.5c14206

**Published:** 2026-02-10

**Authors:** Silvia Pettenuzzo, Marco Roverso, Michele Faralli, Pietro Franceschi, Laura Costantini, Emanuela Betta, Franco Biasioli, Sara Bogialli, Maria Stella Grando, Luca Cappellin

**Affiliations:** 1 Center Agriculture Food and Environment (C3A), University of Trento, San Michele all’Adige 38010, Italy; 2 Research and Innovation Centre, 377369 Fondazione Edmund Mach, San Michele all’Adige 38010, Italy; 3 Department of Chemical Sciences, University of Padova, Padova 35131, Italy

**Keywords:** heat stress, grapevine, metabolomics, volatile organic compounds, physiological response

## Abstract

Grapevine (*Vitis* spp.) is a crop of major economic importance in Europe. Rising temperatures and extreme weather events reduce the yield and alter berry composition, affecting wine quality. Despite advances in vineyard management, mechanisms underlying grapevine thermotolerance remain poorly understood. To fill this gap, we combined physiological and volatile organic compound profiling and untargeted metabolomics. Selected genotypes from a 'Rhine Riesling' × 'Cabernet Sauvignon' progeny were exposed to a 3 h heat shock at 40 and 43 °C under controlled conditions. At 43 °C, genotypes were heat stressed, with a significant chlorophyll fluorescence decrease and modulation of leaf transpiration and metabolism. VOC analysis highlighted 6-methyl-5-hepten-2-one as a candidate heat stress marker, as well as benzenic compounds (i.e., 2-phenyl-2-propanol). Untargeted metabolite profiling revealed several candidate heat-stress biomarkers; notably, the hypothesized 2-*C*-methyl-d-erythritol 2,4-cyclodiphosphate (MEP-cPP) and guanosine accumulated preferentially in susceptible genotypes. Both markers were validated in the field, providing candidate molecular tools for breeding heat-tolerant grapevines.

## Graphical abstract

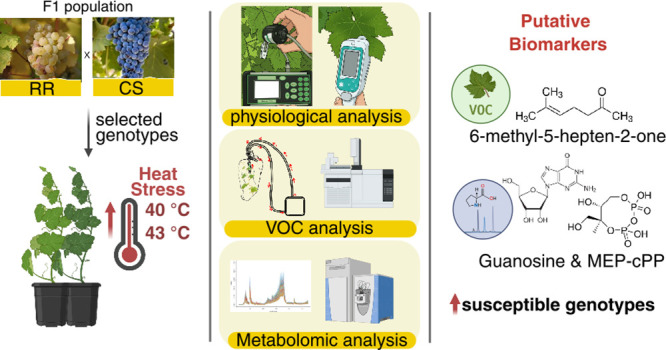

## Introduction

Grapevine (*Vitis* spp.) is the most widely cultivated perennial fruit crop in the world, holding significant economic importance in Europe due to its role in wine production. In recent years, rising temperatures and increased frequency of intense environmental phenomena, such as heat waves, negatively affect grape yield and berry composition with subsequent detrimental effects on wine quality. Even if great efforts have been undertaken to optimize vineyard management strategies and counteract the negative effect of high temperatures,^1^ grapevine mechanisms for thermotolerance are still poorly understood. Heat stress, in fact, is a complex trait to study.^2^ Plants in natural environments are commonly subjected to a great variety of biotic (e.g., pathogens, insects) and abiotic stresses (e.g., drought, salinity, radiation, cold-heat stress), often combined with synergistic, antagonistic, or additive effects depending on the intensity of each stress involved and the order in which they are applied to the plant.^3^ Moreover, plant responses to elevated temperatures are modulated by factors such as age, vigor, and phenological stage, as well as by the intensity and duration of the heat stress.^2^ Therefore, different strategies are likely to be employed by plants to adapt to different stress conditions (e.g., changing leaf orientation, transpirational cooling, alteration of membrane lipid composition).^4,5^ Several phenotypic traits are commonly investigated to assess plant heat stress responses.^6^ Together with physiological parameters, and especially chlorophyll fluorescence,^7^ the profiling of plant metabolites can be used to assess heat stress. Even if metabolites are often acting as nonspecific markers in plants (i.e., phenolic compounds, osmoprotectants, ROS scavengers, amino acids),^8–12^ the presence of specific markers cannot be ruled out. In this type of research, it is important to widen as much as possible the coverage of the plant metabolome relying on “untargeted” metabolomic profiling, which can nowadays be performed thanks to the development of advanced analytical platforms and the increased availability of bioinformatic tools able to manage such complex data sets.

Primary metabolism modulation due to abiotic stresses in plants was reported,^13–17^ and several metabolomic studies related to heat stress on various crops have been already summarized.^18^ Together with primary metabolism, volatile organic compounds (VOCs) have been the subject of specific investigation. VOCs are indeed part of plant secondary metabolites and are implied in various mechanisms of plant interaction with the environment, and in particular, they are modulated in case of abiotic stresses.^19^ Interestingly, Lazazzara et al. reviewed VOCs synthesized by grapevine in response to external stimuli highlighting specific emission patterns depending on the stress applied,^20^ and this observation supports the quest for stress-specific biomarkers. Despite this evidence, investigations on grapevine metabolism modulation in response to increased temperatures are still very few.^21–24^

In this work, grapevine volatile organic compound emission was integrated with a physiological and metabolic investigation. Selected genotypes from a ‘Rhine Riesling’ × ‘Cabernet Sauvignon’ progeny, previously evaluated in the field for physiological traits related to heat stress,^25^ were subjected to heat shock in controlled conditions, along with parental lines. VOC emission was investigated using closed-loop stripping analysis (CLSA), while metabolomic analysis was performed using high-performance liquid chromatography coupled with high-resolution mass spectrometry (UHPLC-ESI-HRMS). Interesting heat stress markers were putatively identified and verified in field conditions, providing a starting point toward understanding differential heat tolerance in grapevine.

## Materials and Methods

### Plant Materials

The study was conducted on selected genotypes of a mapping population obtained from the cross ‘Rhine Riesling’ × ‘Cabernet Sauvignon’ (RRxCS). The cross was made in 2005, and the seedlings were planted in the field on their own roots in 2008. Plants were grown at the Giaroni experimental field of Edmund Mach Foundation (San Michele all’Adige, Trentino Alto Adige, Italy, 46°18′N, 11°13′E) and trained according to the Guyot system.

Woody shoots of parental lines and RRxCS offspring were collected in January 2023 and cut in smaller pieces containing 3 nodes. Only the apical bud was left, and the other two were cut and covered with grafting wax (Fitobalsam, Zapi garden). Cuttings were grown in a climatic chamber at 25 °C with 24 h of light until bud burst and root appearance. Stem cuttings were then transferred in a 13 cm × 9 cm × 9 cm (1 L) plastic container filled with peat moss and grown in greenhouse conditions. Vines were irrigated and fertilized according to greenhouse management and treated against powdery mildew to avoid plant infection. After about two months of growth, cuttings with 12–15 leaves were selected to be studied in a growth chamber. Cuttings of different genotypes were prepared with the same procedure at three different times (January-February-March) to perform the study on plants with the same age.

### In-Field Genotype Selection

Offspring with divergent behavior during hot days across two consecutive seasons (2021–2022) in the field were selected from the whole population, which was characterized for physiological traits at flowering (E-L21, beginning of June), berry pea-size (E-L31, end of June), prevéraison (E-L33, July), and véraison (E-L35, beginning of August). The maximum quantum yield of photosystem II (i.e., F_v_/F_m_) through the measure of chlorophyll fluorescence was used to evaluate plant response to heat stress. Chlorophyll fluorescence transient (O-J–I-P) was measured with a Handy Plant Efficiency Analyzer (Handy-PEA, Hansatech, Norfolk, UK). For each genotype, three measures were taken on the fifth leaf of three different shoots. Leaves were dark adapted for 30 min before measurement. As plants were evaluated in the field, also stomatal conductance (*g*_*s*_), leaf temperature (Tleaf), light-adapted maximum quantum yield of photosystem II (PhiPS2), electron transport rate (ETR), and leaf apparent transpiration (*E*) were assessed via a LI-600 Porometer/Fluorometer (LI-COR Environmental, Lincoln, Nebraska, USA). Measures in nonstress conditions (control, *C*) were taken in the early morning (5:30–8:00), while measures in stress conditions (HS) were collected in the afternoon during the hottest hours (13:00–15:30). Genotypes’ selection was performed by evaluating the results of principal component analysis (PCA). For this purpose, the difference between stress and nonstress conditions of measured parameters was calculated using the following formula: Δ% = [(*C* – HS)/*C*] × 100.^25^

### Controlled-Environment Conditions: Experimental Design and Heat Treatment

Stem cuttings of each genotype were acclimated for 5 days in a controlled environment (CLIMACELL-E-707-c, MMM Group, Munich, Germany) with 70% ± 5% of relative humidity (RH). Temperatures were set at 25/20 °C day/night, with a photoperiod of 16 h under white fluorescent light at 100 μmol m^–2^ s^–1^ plus red/blue LED light at 600 μmol m^–2^ s^–1^. Plants were irrigated to full pot capacity on alternate days during the acclimatation period and after the stress application. Chamber temperature and humidity were monitored with a data logger during experiments. After the acclimatation period, plants were subjected to a sudden heat shock (HS). The temperature of the chamber was raised to 40 or 43 °C in 30 min and maintained for 3 h (from 11:00 to 14:00). After the heat shock, the temperature was set back to 25 °C for recovery. For each genotype, two identical but distinct experiments were performed with different biological replicates due to analysis incompatibility. In particular, one experiment (*N*= 5 biological replicates) was performed to sample the volatile organic compounds emitted by plants with CLSA, while physiological measurements and leaf sampling for metabolomic analysis were performed in a second experiment (*N*= 6 biological replicates). Measurement of physiological parameters, VOCs, and leaves sampled before the stress application were considered as control. VOC collection with the CLSA technique was then performed during 3 h of heat shock, while leaves were sampled immediately after the end of the treatment. Physiological parameters were measured during stress application, at the end of the temperature ramp and after 1, 2, and 3 h of heat shock, and during recovery step (1–3 h after the end of the heat shock and after 24 h). To confirm results obtained in controlled conditions, leaves of selected genotypes of the population were sampled in the field to perform metabolomic analysis in two different seasons (2022 and 2023).

### Controlled-Environment Conditions: Physiological Evaluation

Chlorophyll fluorescence transient (O-J–I-P) was measured with a Handy-PEA. Measurements were performed on three leaves for each of the six biological replicates. The third, sixth, and ninth leaves starting from the cutting base were measured at the different time points reported in the above section. Before measurement, each leaf was dark adapted for 25 min with suitable leaf clips. The instrument was set up with a gain of 0.9 and a saturating pulse of 3500 μmol m^–2^ s^–1^. Leaf transpiration parameters were measured with the LI-600 Porometer/Fluorometer. “Auto gsw” was selected as a protocol for LI-COR measurements.

### Reagents

All reagents and solvents were of HPLC or LC-MS grade. Ultrapure water was obtained with a Purelab Chorus system (18.2 MU cm) (ELGA LabWater, High Wycombe, United Kingdom). Acetonitrile, dichloromethane, formic acid (FA), alpha-copaene (97%), beta-ocimene (90%), beta-myrcene (95%), gamma-terpinene (97%), beta-caryophyllene (98%), farnesene (isomers mix), gamma-bisabolene (97%), germacrene D (98%), nerol (97+%), nerolidol (98%), linalool (97%), linalool oxide (97%), beta-cyclocitral (98%), methyl salicylate (98%), alpha-ionone and beta-ionone (90%), geraniol (98%), transcarveol (95%), acetophenone, 1-decanol, and 2-octanol were supplied by Merck. Phenylalanine-d_5_, used as internal standard (IS), was purchased from LGC-standard (Teddington, United Kingdom).

### Volatile Organic Compound Analysis

#### CLSA VOC Sampling and Extraction

Volatile organic compounds were sampled under controlled conditions with the CLSA technique.^26^ Each plant (*N* = 5) was enclosed in a plastic bag (Cuki oven bag, Cofresco, Volpiano, Italy), previously conditioned at 65 °C for 3 days to reduce bag emissions to minimum. VOCs were also sampled in an empty bag as a negative control. VOC samples were collected using an adsorbent filter (glass tube, 6.5 × 0.55 × 0.26 cm, loaded with 1.5 mg of activated charcoal; CLSA filter LR-type; Brechbühler AG, Schlieren, Switzerland) connected to a 12 V rotary pump (Fürgut, Tannheim, Germany) with a short Teflon tube. Pumps were connected to 6 V rechargeable sealed lead batteries (Energy Safe, SGR group, Milan, Italy) with a flow rate of 2 L/min. VOCs were sampled for 3 h the day before stress application (C) and during the heat shock (HS) from 11:00 to 14:00. As volatile organic compound emissions are known to be regulated by circadian rhythms, sampling with CLSA was performed for each parental variety and offspring genotype at the same hours of the day, from 11:00 to 14:00, which is the time at which plants normally experience the highest radiation and increasing temperature in natural conditions. Moreover, as reported by Giacomuzzi et al., grapevine leaf daily rhythm of VOC emission does not change significantly during the day, while it is influenced by changes between day and night.^27^ Therefore, the only contribution studied in this experiment should be the temperature effect. Samples were eluted from the filter with a double elution of 100 μL of dichloromethane each, 10 μL of 2-octanol (IS, 100 mg/L) was added, and then samples were stored at −80 °C.

#### GC-MS Analysis

Samples (1 μL) were analyzed in random order with a Clarus 500 GC-MS (PerkinElmer, Waltham, Massachusetts, USA) equipped with an autosampler (Cycle Composer, PAL system, CTC analytics, Zwingen, Switzerland) and a polar column HP-Innowax (30 m × 0.32 mm × 0.50 μm, Agilent Technologies, Santa Clara, USA). Helium was used as a carrier with a flow rate of 1.5 mL/min. The analyses were carried out with a temperature ramp: the starting temperature (40 °C) was held constant for 4 min, followed by an increase of 2 °C/min until 60 °C was held constant for 1 min. The temperature was increased again with a rate of 5 °C/min until 190 °C held constant for 1 min and then increased to 230 °C with a rate of 10 °C/min and then kept constant for 4 min.^28^ Samples were injected in split mode (50:1) with an inlet temperature of 250 °C and 4 min of solvent delay. The mass spectrometer, equipped with an electron impact ionization source operating at 70 eV, was set to scan from *m*/*z* 33 to 350.

### Metabolomic Analysis

#### Leaf Sampling and Extraction

For metabolomic analysis, the second, fifth, and eighth leaves of each biological replicate were collected as control samples before the start of the heat shock, while the fourth, seventh, and tenth leaves were collected as stress samples at the end of the 3 h of heat shock. Leaf sampling in the field during hot days was performed on the sixth leaf on the three shoots between 5:30 and 8:00 in the morning as control, while the seventh leaf was sampled during heat stress between 13:00 and 16:00. Leaves were immediately frozen in liquid nitrogen and subsequently stored at −80 °C.

Frozen leaf powder (200 mg) was extracted with 1 mL of a methanol:ultrapure water (70:30) solution containing phenylalanine-d_5_ added as surrogate IS with a concentration of 200 μg/L. The extraction mixture was sonicated for 15 min and then centrifuged for 10 min at 14000 rpm. The supernatant was then lyophilized and resuspended in 200 μL of ultrapure water with 0.1% formic acid. Quality control (QC) samples were prepared from a mixture of frozen leaf powder of all of the genotypes under analysis at control and stress and extracted with the same protocol. Samples were prepared in a random order.

#### LC-HRMS Analysis

Leaf extracts (2 μL) were analyzed in random order with an Ultimate 3000 UHPLC chromatograph coupled with a Q-Exactive hybrid quadrupole-Orbitrap mass spectrometer (Thermo Fisher Scientific). An Acclaim mixed-mode WCX-1, 3 μm, 120 Å, 2.1 mm × 150 mm (Thermo Fisher Scientific) column thermostated at 20 °C was used. Eluents were ultrapure water and acetonitrile, both acidified with 0.1% formic acid, at a flow rate of 0.2 mL/min. The gradient was 100% water for 3 min, linearly increasing to 100% acetonitrile in 15 min, 100% constant acetonitrile for 4 min, and then 100% water in 1 min, equilibration for 7 min. Mass spectrometric conditions were as follows: electrospray ionization (ESI) in both positive (+) and negative (−) modes, data dependent acquisition with resolution 35,000 in full MS and 17,500 in MS/MS, AGC target 1 × 10^6^ in full MS and 1 × 10^5^ in MS/MS, max injection time of 100 ms, *m*/*z* range 70–1000, isolation window 2.0 *m*/*z*, collision gas nitrogen, normalized collision energy 25 eV. Data dependent settings were as follows: minimum AGC target 9 × 10^3^ and dynamic exclusion 20 s. The capillary voltage was 4.0 kV (+)/ 2.8 kV (−), the capillary temperature was 300 °C (+)/280 °C (−), and auxiliary gas was nitrogen at 20 psi. Calibration was performed with a standard solution (Pierce LTQ Velos ESI positive/negative ion calibration solution, Thermo Fisher Scientific), and Xcalibur 3.1 software was used for instrument control (Thermo Fisher Scientific).

### Data Processing

Both GC-MS and LC-MS raw data were converted to ABF format (Reyfics Analysis Base File Converter) and processed with MS-DIAL 4.9, an open access software for feature extraction, grouping, alignment, and identification (http://prime.psc.riken.jp/compms/msdial/main.html).^29^ Parameters for data extraction were selected on the basis of the instruments and chromatographic performances. They are reported in detail in Tables S1 and S2 (Supporting Information). For GC-MS data, among the features detected with MS-DIAL, only compounds showing a concentration significantly different from that measured in blanks were considered for further investigations. Volatile compounds were identified by comparing experimental mass spectra with the MS-DIAL GC-MS reference library “All records with Kovats RI” in the NIST MSP format. The identification was confirmed by comparing mass spectra with data available in the NIST reference library (NIST 08, National Institute of Standards and Technology, Gaithersburg, MD, 2008) and their Kovats retention indices (RIs), experimentally determined, with RI described in the literature. When available, the identification was further verified with injection of the analytical standard. Among the features detected with MS-DIAL, statistically different from blanks (88 features), 52 compounds were correctly extracted with an informative EI mass spectrum, and 28 were identified (Table S3, Supporting Information).

Preprocessing of LC-MS data resulted in 4185 features in positive and 8057 in negative ionization mode. Peak areas of each feature were normalized by internal standard (phenylalanine-d_5_, relative standard deviation (RSD%) = 2–20%) and QCs (using the LOESS normalization), with the function available in MS-DIAL. Data were further normalized by subtracting at each feature the mean of the genotype to reduce the variability related to varietal abundance and highlight differences related to the application of the heat shock. Feature annotation was tentatively performed by comparing experimental tandem mass spectra with MS-DIAL LC-MS reference libraries “ESI­(+)-MS/MS from authentic standards (16,481 unique compounds)” and “ESI(−)-MS/MS from authentic standards (9,033 unique compounds)”. Unknown compounds of interest were analyzed with MS-FINDER 3.5, an open access structure-elucidation program that allows the annotation of unknown compounds by predicting their elemental formula and by comparing the experimental tandem mass spectra with metabolome databases and an *in silico* mass spectral fragmentation (http://prime.psc.riken.jp/compms/msfinder/main.html). When standards were available, retention times and tandem mass spectra of compounds present in samples were compared with those of the standards injected under the same conditions.

For GC-MS data, peak areas of each compound were normalized by internal standard (RSD% = 5–17%), with the function available in MS-DIAL, to compensate for possible different extraction yields among samples and instrumental drifts during analysis. An additional normalization for *g_s_* was then applied to compare parental lines and progeny individuals. As environmental conditions like temperature and light availability have an influence in the opening and closure of stomata, which can then modify VOC intercellular partial pressure, in non-steady-state conditions, stomata can exert a certain control in VOC emission.^30^ Due to the rapid changes in temperature applied in this experiment, it was assumed that plants inside the binder were subjected to non-steady-state conditions, and consequently, the normalization for *g_s_* was applied. For each genotype, areas of each feature at control were normalized for the corresponding mean stomatal conductance (*N* = 6, measured in the second experiment). The same approach was used for data collected in stress conditions, in which the mean stomatal conductance (*N* = 6) of the 3 h of stress was used for normalization.

### Statistical Analysis

Multivariate analysis, statistical analysis, and graphical representation were performed using R software environment 4.2.1 (https://www.r-project.org/). For Principal component analysis (PCA), the following packages were used: “ggfortify”^31^ and “ggplot2”.^32^ Correlations were calculated with the package “tidyverse’”^33^ For the analysis of plant physiological responses, data for each genotype were normalized to the corresponding maximum value observed under the control conditions and expressed as percentages to allow comparison among genotypes. To assess the significance of differences among experimental conditions during shock application (control, stress, and recovery), we further normalized data within each condition to the mean value of the parameter, minimizing the contribution of biological replicate variability. A linear model was then fitted to the normalized data, and predicted values and confidence intervals were estimated using the “stats”^34^ and “marginaleffects”^35^ packages.

Due to the non-normal distribution of data, Spearman correlations were calculated. The *t* test and one-way ANOVA (ANOVA) were performed with the packages “rstatix”^36^ and “devtools”.^37^ Effect sizes were calculated and plotted with packages “effectsize” and “effsize”.^38^ Graphical representations were performed with the package “ggplot2”. Gene Ontology analysis for metabolomics was performed using INCHIKEY codes of identified metabolites on the IDSL.GOA web site (https://goa.idsl.me/).

## Results and Discussion

### In-Field Genotype Selection

Principal component analysis of data collected in the field in 2021 and 2022 at different phenological stages shows similar results between vintages, with minimal changes in the position of some genotypes depending on the phenological stage and year. The PCA of measurements taken at berry pea size in 2022 is reported in Figure 1. PC 1 explains the difference in chlorophyll fluorescence parameters among the genotypes. Moving from left to right in PC 1, genotypes have a higher decrease in F_v_/F_m_ and Phi­(Eo). On the other hand, PC 2 explains the difference in transpiration. Going from top to bottom in PC 2, genotypes tend to have more open stomata during stress. Genotypes that have a higher decrease in F_v_/F_m_ tend to have a higher leaf temperature during stress, although this is not true for all the genotypes of the progeny (e.g., genotype 34). Furthermore, the increased transpiration during the stress of some genotypes is not always matched by a reduced leaf temperature.

**Figure 1:**
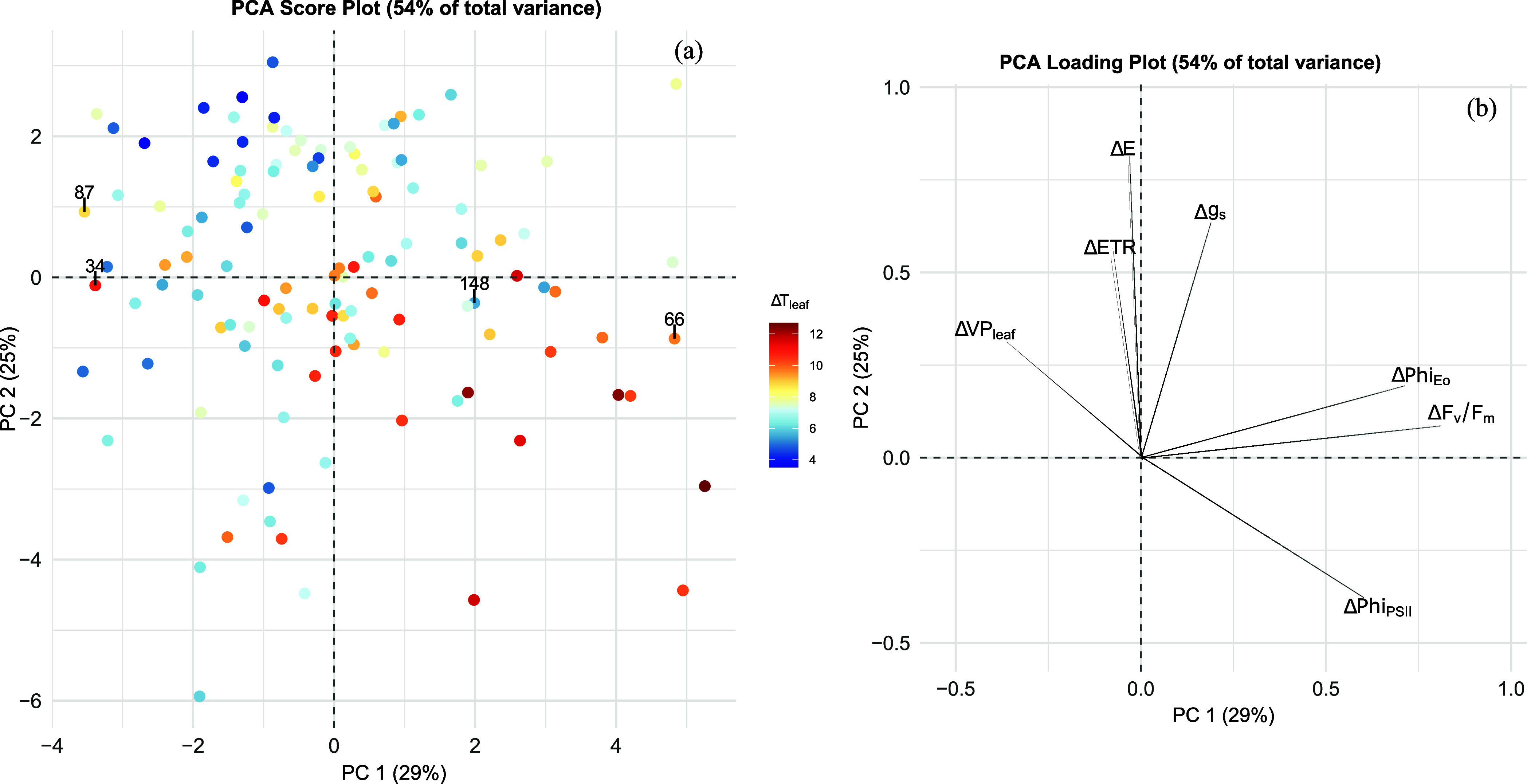
Score plot (a) and loading plot (b) obtained from the principal component analysis (PCA) of physiological variables measured in the June 2022 sampling session (Berry pea-size 2022). Components 1 and 2 were rotated with the “principal” function of the package “Psych” in R. Genotypes are reported as dots, and colors are based on the difference in leaf temperature between control and stress (color scale ranges from blue (Δ*T* = +4 °C) to red (Δ*T* = +12 °C) with the increase in temperature differential). Genotypes selected for the experiment under controlled conditions are highlighted with their numerical code.

Based on the results of the PCAs, four genotypes were selected for further analysis under controlled conditions along with parental lines: genotypes 34, 66, 87, and 148. Genotypes 34 and 87 were selected due to their F_v_/F_m_ (Table 1) and their transpiration being unaffected despite an increase in their leaf temperature of ∼10 °C in the afternoon. On the contrary, genotype 66 showed the highest decreases in F_v_/F_m_ and its behavior was consistent through all the phenotyping sessions despite maintaining stomatal opening (Table 1). Genotype 148 was selected, as it showed a decrease in F_v_/F_m_ of almost 10% in most of the phenotyping sessions, with a minimum increase in leaf temperature. As highlighted in Figure 1, these four genotypes are also well distributed along the direction of largest variability in the data set (PC 1).

**Table 1:**
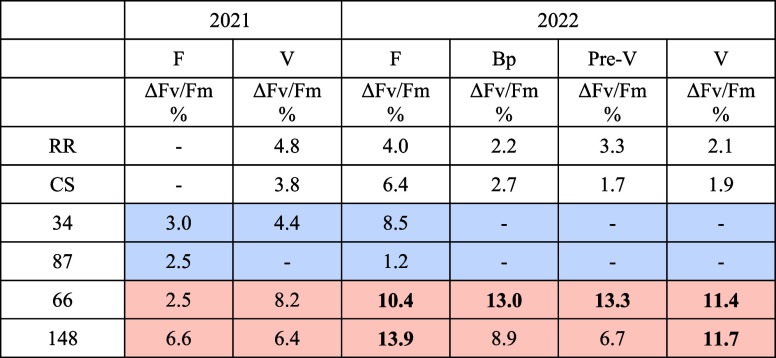
Percentage Values of the Chlorophyll Fluorescence Decrease (ΔF_v_/F_m_ %, Normalized for Control Values) at Different Sampling Sessions (F = Flowering, Bp = Flower Pea Size, Pre-V = Pre-véraison, V = V = V) for Parental Lines and Genotypes Selected for the Experiment in Controlled Conditions^a^

a Parental lines are reported with the code RR= Rhine Riesling and CS = Cabernet Sauvignon. Genotypes that maintain their F_v_/F_m_ during hot days are highlighted in light blue, while genotypes that decrease their F_v_/F_m_ are highlighted in orange. Differences in F_v_/F_m_ > 10% are highlighted in bold, and differences <1% are reported with (−).

To confirm the results obtained under controlled conditions, leaves of parental lines and selected genotypes of the progeny were sampled in the field to perform metabolomic analysis in two different seasons (2022 and 2023). In the first season, together with genotypes selected for the experiment under controlled conditions, other 5 genotypes were included in the metabolomic analysis based on their physiological response in the field (in season 2021 and 2022 up to prevéraison): genotypes 101 and 188 were selected as presumed tolerant for the maintenance of F_v_/F_m_ near the control values in the afternoon, while genotypes 8, 195, and 211 were selected as presumed susceptible. In the second season, the number of selected genotypes was further increased to a total of 10 genotypes chosen as “susceptible” (coded as 32, 85, 158, 185, 193, 251) and 13 as “tolerant” (coded as 20, 56, 90, 112, 124, 145, 161, 249) based on their physiological response in the field (season 2021–2023). The values of maximum quantum yield of photosystem II (PSII) decrease (ΔF_v_/F_m_ %, normalized for control values) for selected genotypes are reported in Table S4, Supporting Information.

### Heat Shock at 40 °C

According to Venios et al., the activation of heat acclimation mechanisms in the photosynthetic apparatus occurs at temperatures above 35 °C, whereas photosynthetic activity becomes severely impaired at temperatures exceeding 40 °C.^12^ Based on previous heat stress studies on grapevine,^39–41^ in which typical stress temperatures are comprised between 40 and 45 °C, and on preliminary experiments performed on Cabernet Sauvignon stressed at different temperatures (39–45 °C for 3 h, data not shown), plants were initially subjected to heat shock at 40 °C. Results of this preliminary experiment highlighted a similar reduction in F_v_/F_m_, between 1 and 9.5% compared to control, for all genotypes analyzed. Moreover, photosynthesis recovery started before the end of heat shock application, reaching a level comparable to the control after the first hour of recovery (Table S5, Supporting Information).

As far as metabolomic analysis is concerned, although the reduction in F_v_/F_m_ was significant for certain genotypes (e.g., such as Cabernet Sauvignon), heat stress did not lead to a notable change in the plant primary metabolism. Indeed, while *t* tests identified some significant features (*P* < 0.05) in both positive and negative ionization modes, P value distribution plots showed a uniform distribution across all analyzed genotypes, indicating no consistent metabolic modulation. At odds with primary metabolism, significant modulation of VOCs was observed for CS. In particular, CS emission of terpenoids increased (*P* < 0.05, effect size >0.5), like beta-myrcene, gamma-terpinene, alpha-terpineol, beta-cubebene, gamma-muurolene, alpha-copaene, and isocitral. Nonetheless, compounds with a major increase compared to control were 2-phenylethanol, benzyl alcohol, and 2-nonanone. This trend was visible also for the other genotypes, even though not statistically significant (data not shown). These findings suggest that a 3 h heat shock at 40 °C is not sufficient to cause a significant modulation of grapevine primary metabolism, with only VOCs and the maximum quantum yield of PSII mildly affected (ΔF_v_/F_m_ < 10%), as all the genotypes recover quickly before the end of the shock application. For these reasons, a higher temperature was selected to perform a second study. The choice of 43 °C was based on results obtained from the Cabernet Sauvignon preliminary heat shock study (data not shown). In fact, 43 °C caused a significant decrease in F_v_/F_m_ compared to the control, while maintaining a smaller variance between biological replicates compared to 45 °C.

### Heat Shock at 43 °C

#### Physiological Response

When heat shocked at 43 °C, RR and CS showed similar physiological responses. In both varieties, physiological parameters tended to decrease significantly their values after 1 h of heat shock, compared to control (Figure 2), with a maximum reduction in F_v_/F_m_ (% to control) of 16.1 and 16.6%, respectively, after 2 h of heat shock (Table 2). At the end of the stress, CS recovered slightly more quickly, with F_v_/F_m_ returning to a control level after 2 h at 25 °C, while RR was recovered after 3 h.

**Figure 2:**
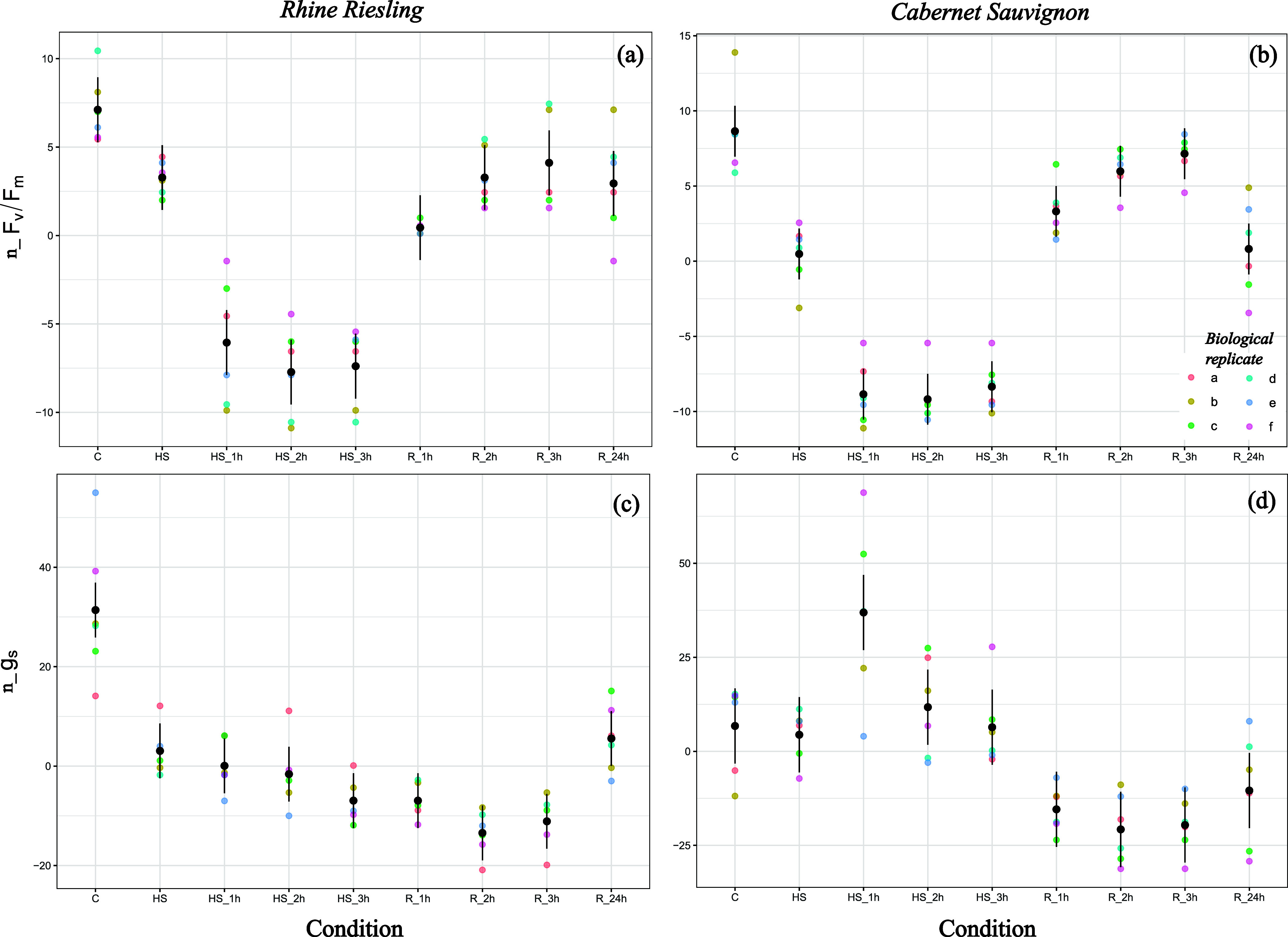
Rhine Riesling and Cabernet Sauvignon physiological response when heat shocked at 43 °C for 3 h (*N* = 6). Colored dots show the trend of F_v_/F_m_ and *g_s_* measured at control (*C*, *T* = 25 °C), at the reach of 43 °C (HS), after 1–3 h of heat shock (HS_1h, HS_2h, HS_3h), and at recovery after 1–24 h since the end of the heat shock (R_1h, R_2h, R_ 3h, R_24h). For each condition, data were normalized by the maximum value obtained at control (see Figure S1, Supporting Information) and by the mean value of the parameter. Results of the linear model, expected values (dots) and confidence intervals (lines), are reported in black.

**Table 2:**
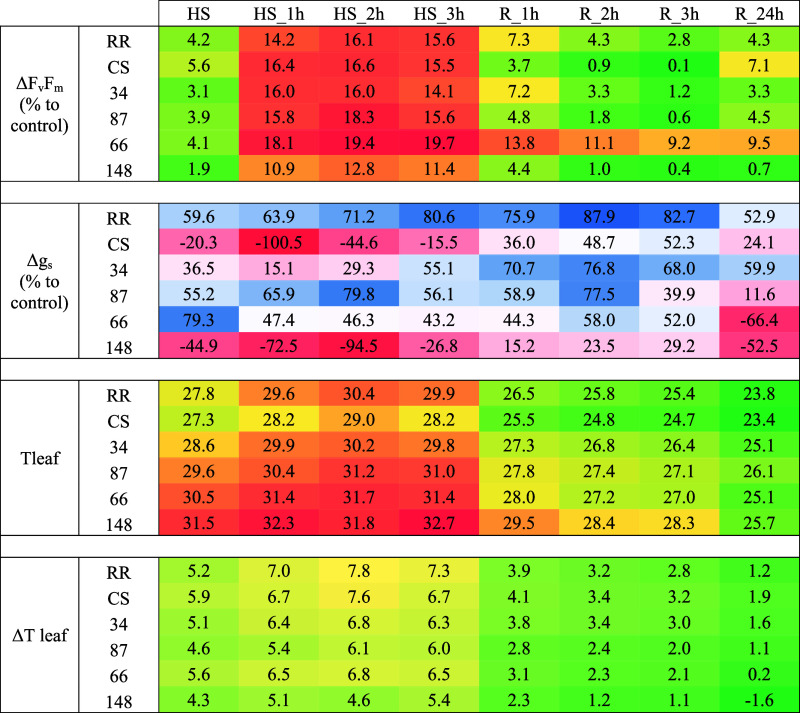
Physiological Response of Genotypes When Heat Shocked for 3 h at 43 °C^a^

a ΔF_v_/Fm and Δ*g_s_* values were calculated as (*C* – *X*)/*C* × 100, while Δ*T* leaf was calculated using (*X* – *C*), where *C* is the value at control and *X* is the value either at 43 °C or after 1–3 h of stress or after 1–24 h of recovery. For each genotype at different time points is reported the mean value of *N* = 6 biological replicates. Color codes are as follows: from green to red for increasing differences in F_v_/F_m_ and increasing leaf temperatures; from green to yellow for increasing differences in leaf temperatures; from blue to red for *g_s_* variations, from stomata closure (blue) to stomata opening (red).

Parental lines showed opposite behaviors under heat shock for *g_s_* responses. Indeed, RR closed its stomata when heat shock began, with a decrease of *g*_*s*_ of 60 to 80% (compared to control) during the 3 h of heat shock (see Table 2). Stomatal conductance did not return to control levels even after the end of the stress and rewatering (Figure 2b). On the contrary, CS tended to open its stomata during stress, with *g_s_* returning to control levels after heat shock. This behavior was reflected in leaf temperature; by opening the stomata, in fact, CS maintained a leaf temperature approximately 1.5 °C lower than RR (see Table 2). Despite this, CS exhibited a slightly greater decrease in F_v_/F_m_ (% of control), especially at 43 °C—5.6% in CS compared to 4.2% in RR—and after the first hour of stress, with CS F_v_/F_m_ decreasing by 16.2% versus 14.2% in RR (Table 2).

In the progeny, genotypes 34 and 87 were selected for the capacity of maintaining F_v_/F_m_ and *g*_*s*_ at control levels during hot days, despite the increase in leaf temperature. When heat shocked at 43 °C in controlled conditions, F_v_/F_m_ tended to decrease in both genotypes with a behavior similar to that of RR. In fact, when the temperature reached 43 °C, the decrease in F_v_/F_m_ (% to control) was not significant: 3.1% for genotype 34 and 3.9% for genotype 87 (Table 2). On the other hand, genotypes were significantly stressed after 1 h at 43 °C, with a reduction in F_v_/F_m_ of 16% compared to control values, like RR. Together with the reduction in F_v_/F_m_, genotype 34 showed similar leaf temperature to RR during stress (Table 2), with an increase of up to 7 °C compared to control values. Conversely, genotype 87 experienced a reduction in F_v_/F_m_ of 18% after the second hour of heat shock, with a leaf temperature of 31 °C (+6 °C compared to control values). Following heat shock, F_v_/F_m_ returned to control values after 3 h of recovery for genotype 34, while genotype 87 was fully recovered already after 2 h (Figure 3a,c), both being slightly quicker compared to RR. By contrast, the two genotypes differed for their *g_s_*. In fact, genotype 34 seems to have a slow response to heat shock for stomata opening/closure. Indeed, *g*_*s*_ was almost unaffected during heat shock, and it decreased significantly up to 77% only at the end of it, not recovering in 24 h at 25 °C (see Table 2), while genotype 87 did not show a significant difference between stress and control conditions (Figure 3b,d).

**Figure 3:**
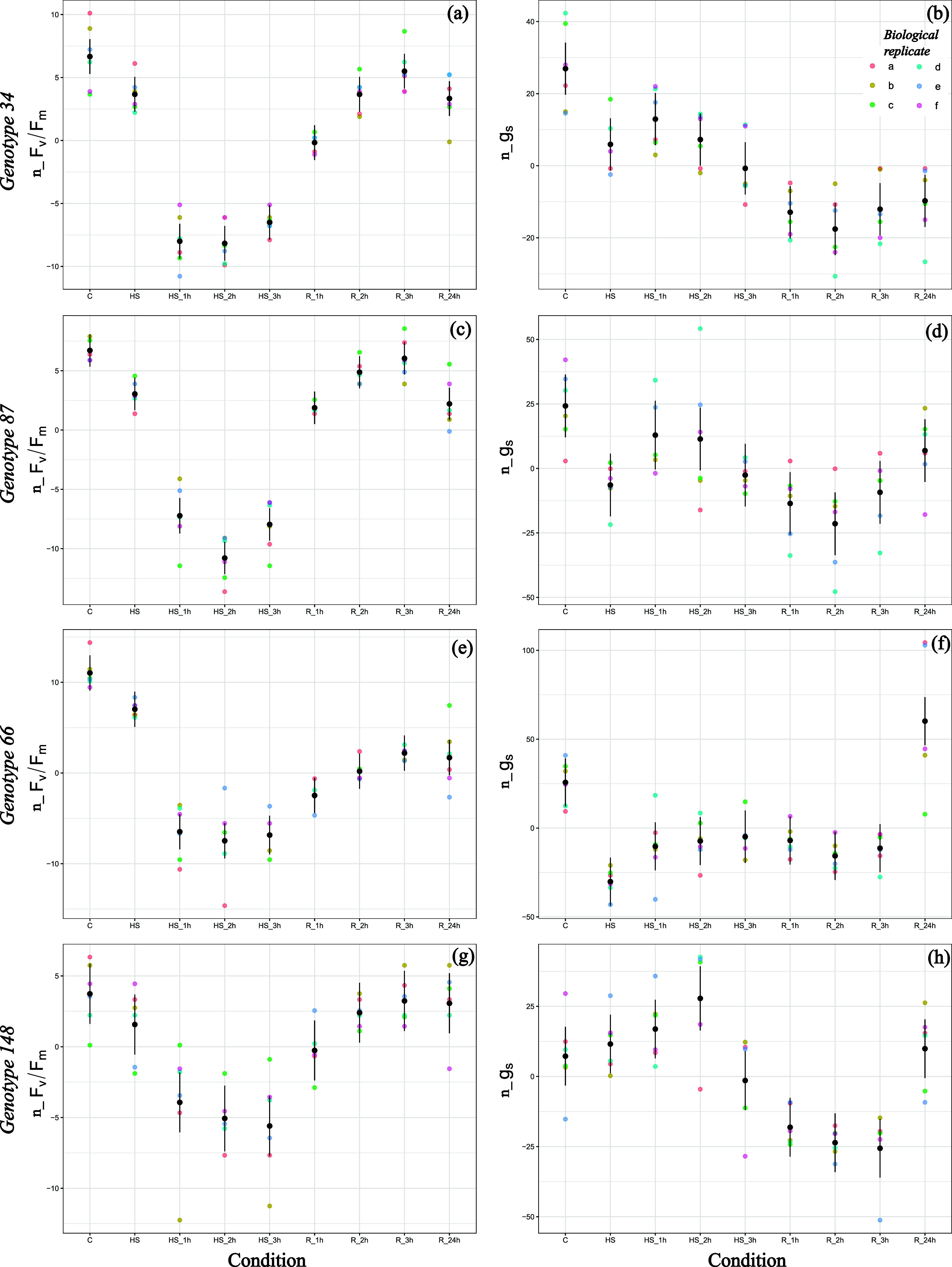
Physiological response of genotypes 34, 87, 66, and 148 when heat shocked at 43 °C for 3 h (*N* = 6). Colored dots show the trend of Fv/Fm and *g_s_* measured at control (*C*, *T* = 25 °C), at the reach of 43 °C (HS), after 1–3 h of heat shock (HS_1h, HS_2h, HS_3h), and at recovery after 1–24 h since the end of the heat shock (R_1h, R_2h, R_ 3h, R_24h). For each condition, data were normalized values by the maximum value obtained at control (see Figure S2, Supporting Information) and by the mean value of the parameter. Results of the linear model, expected values (dots) and confidence intervals (lines), are reported in black.

Conversely, genotypes 66 and 148 were selected among the progeny because of the decrease in their quantum yield of PSII during hot days, which was not only higher compared to the average response of the population but also consistent throughout the years and phenological stage, especially for genotype 66. The behavior of this genotype was confirmed also in controlled conditions. When heat stressed at 43 °C, in fact, F_v_/F_m_ decreased of 18.1–19.7% (after 1 and 3 h of heat shock, respectively), and it never returned to control values even after 24 h of recovery (Figure 3). Moreover, genotype 66 had one of the highest leaf temperatures during stress (∼32 °C; Table 2). Looking at stomatal conductance and leaf transpiration behavior, genotype 66 had a peculiar response. In fact, *g*_*s*_ decreased significantly upon heat shock beginning, similar to what happened for RR, but then, after 24 h of recovery, it was back to control values. On the other hand, genotype 148 behavior is difficult to interpret. During heat shock, in fact, the decrease in Fv/Fm is significant after 1 h of stress (Figure 3g), but the decrease is smaller compared to the other genotypes (Table 2). Moreover, as CS, genotype 148 tended to open its stomata and increase its transpiration rate (Table 2). Despite that, its leaf temperature during stress was even higher than that registered for genotype 66, reaching 32.7 °C after 3 h of heat shock (Table 2).

Thus, from a physiological perspective, it appears that at 43 °C, all genotypes analyzed experience an F_v_/F_m_ reduction between 16 and 20%. For 5 genotypes out of 6 analyzed, physiological behavior predicted from field data was confirmed in controlled conditions. Genotypes selected as “tolerant” in the field (34, 87, and parental lines), in fact, showed a lower decrease in F_v_/F_m_ compared to genotype 66, selected as “susceptible”, and a faster recovery at the end of the shock application. Under these experimental conditions, CS appears to be slightly more affected by heat shock compared to RR, as evidenced by its decrease in Fv/Fm despite the opening of the stomata and the lower leaf temperature. However, it should be noted that RR recovered more slowly after the stress application. Our findings support those of Hewitt et al., who studied RR and CS under drought and heat stress, focusing on the transcriptome and metabolome of grape berries.^42^ Thanks to transcriptomic analysis, they proposed different genetic regulation mechanisms for the two varieties, which ultimately exhibit similar berry metabolic regulation and comparable physiological responses.

#### VOC Emission Profile

The PCA of the data shows the difference between control samples (blue, Figure 4a) and stressed samples (red). In particular, PC 1 highlights the separation of genotype 66 emissions compared to other genotypes and parental lines, suggesting and confirming that genotype 66 shows a peculiar behavior when subjected to heat shock. On the other hand, the score plot obtained from PC 2 and 3 (Figure 4b) shows differences between control and stressed samples of other genotypes, especially CS, highlighting 87 and 148 as genotypes with the smallest variance between the two conditions.

**Figure 4:**
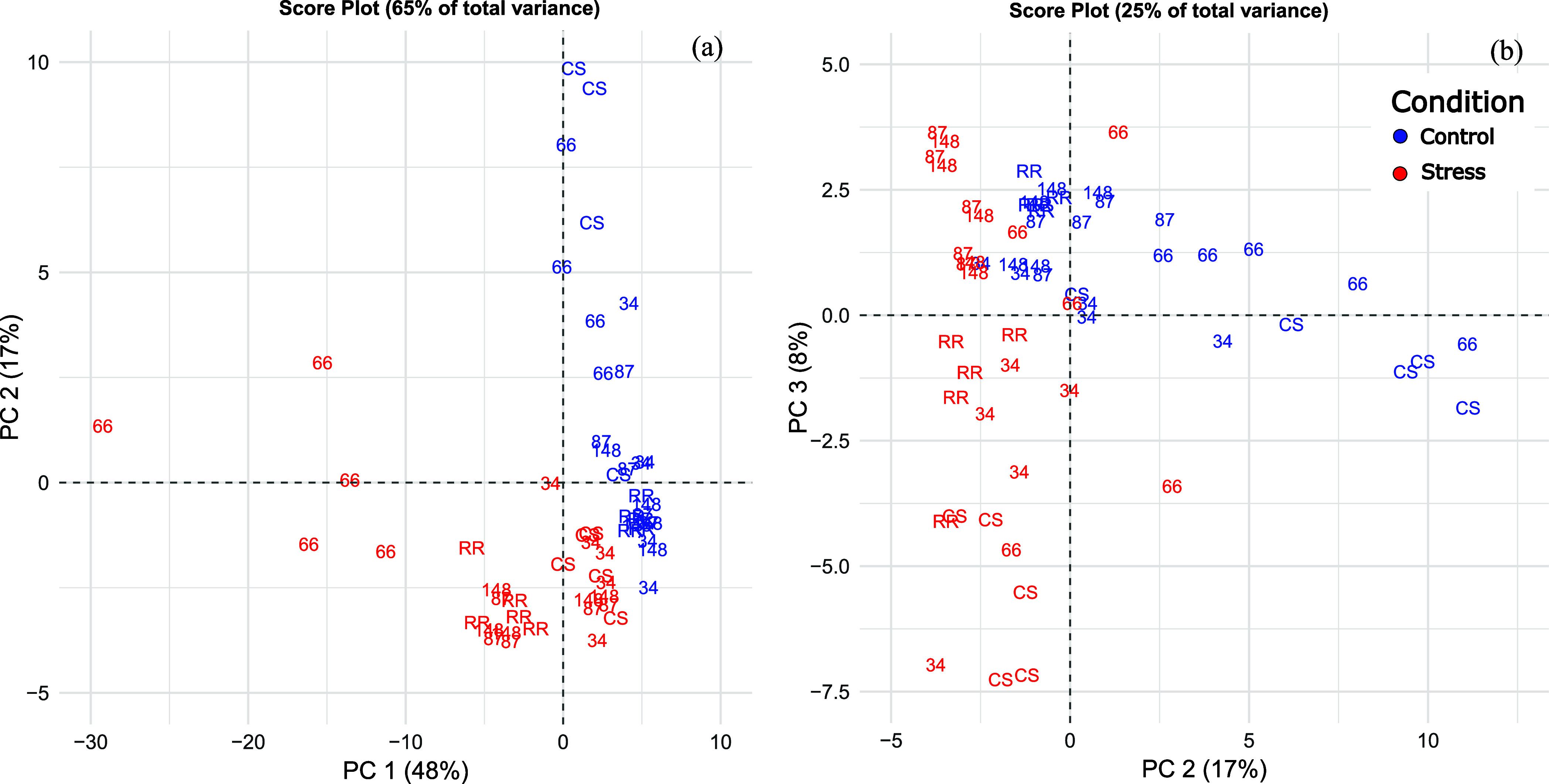
Score plots of components 1 and 2 (a) and components 2 and 3 (b) of the principal component analysis for the CLSA results obtained from the VOC analysis of the biological replicates of RR, CS, and genotypes 34, 66, 87, and 148 at control 25 °C (blue) and during the application of the heat shock at 43 °C (red).

The *t* tests performed for each compound show a statistically significant difference for all the genotypes and parental lines between stress and nonstress conditions. In particular, for all the genotypes analyzed, there was a decrease in the emission of monoterpenes such as beta-myrcene and beta-ocimene (*P* < 0.05, effect size > 0.5) during heat shock (see Figure 5).

**Figure 5:**
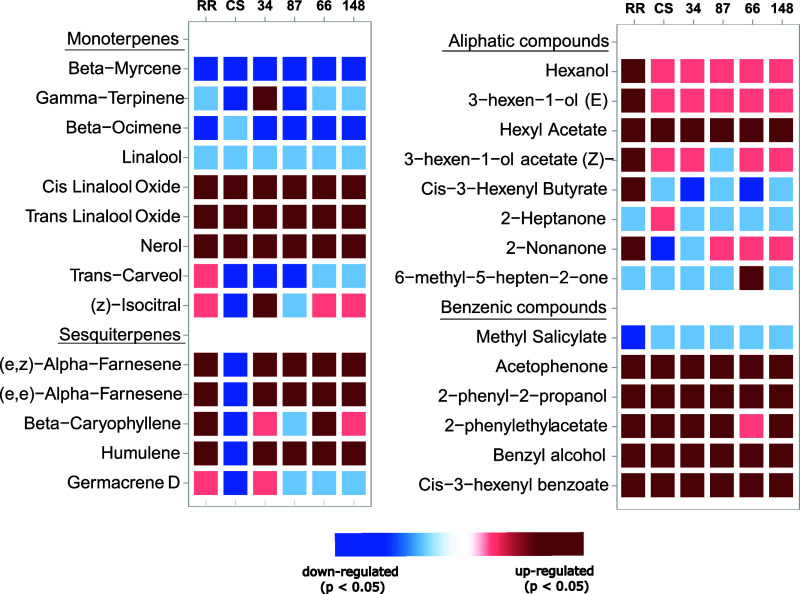
Heat shock-induced modulation of VOCs for genotypes studied in this experiment (with genotypes 34 and 87 presumed tolerant and genotypes 66 and 148 presumed susceptible). VOC modulation was calculated using (HS – *C*)/*C* × 100, where HS is the area of the metabolite in the stressed sample (43 °C) and *C* is the area of the metabolite at control. In dark red and blue, VOCs that are upregulated and downregulated during stress (*P* < 0.05 and effect size > |0.5|), respectively; in light red and blue, metabolites that have a tendency to increase or decrease with heat shock but with *P* > 0.05, respectively.

For CS and genotype 87, gamma-terpinene, isocitral, and *trans*-carveol were also emitted in lower quantities during heat shock at 43 °C, together with the sesquiterpene germacrene-D. Additionally, only for CS, other sesquiterpenes were emitted in lower quantities during heat shock: caryophyllene, humulene, (*E*,*E*)-alpha-farnesene, and (*E*,*Z*)-alpha-farnesene. This behavior was opposite to that observed when heat shocked at 40 °C, in which an increased emission in terpenoids was observed. These sesquiterpenes were, on the other hand, more emitted in stress conditions for RR, genotypes 34, 66, and 148 (Figure 5). Emission of 6-methyl-5-hepten-2-one was lower during heat shock for all the genotypes analyzed except for genotype 66 for which a higher emission was registered. Conversely, compounds that were emitted in higher quantities during stress conditions in all genotypes analyzed are *cis*- and *trans*-linalool oxides and nerol for the class of terpenoids; acetophenone, *cis*-3-hexenyl benzoate, 2-phenyl-2-propanol, and benzyl alcohol for the class of benzenic compounds; and hexyl acetate for aliphatic compounds (see to Figure 5). Hexanol, (*Z*)-3-hexenol, 3*E*-hexenyl acetate, and *cis*-3-hexenyl butyrate were higher during stress only for RR, while 2-phenylethyl acetate emission was higher in the stress condition for all genotypes except for 66, for which the difference between control and stress was not significant.

The obtained results suggest that grapevine modifies its VOC emission when subjected to a heat shock, and plants tend to emit benzenic compound derivatives, such as benzyl alcohol, 2-phenylethanol, and 2-phenylethyl acetate, in response to a temperature shock. These compounds, in fact, are not emitted or emitted in low quantities at the optimum temperature (data not shown). Benzenic compound derivatives have been previously reported as related to grapevine response to both biotic and abiotic stresses, as described by Lazazzara et al.,^20^ but no relation to high temperatures was found in grapevine until 2022. Campos-Arguedas et al., in fact, reported an accumulation of 2-phenylethanol and other benzenic compounds in grape berries upon increasing temperatures.^22^ These findings support the results obtained in our experiment, which refer for the first time to grapevine leaves. Additionally, our work pointed out a peculiar behavior for terpenoid emission. In fact, with a 40 °C heat shock, all genotypes with the exclusion of CS showed an increased emission in monoterpenes and sesquiterpenes, which became statistically significant when plants were heat shocked at 43 °C. At odds, for CS, the increase was statistically significant already at 40 °C, while at 43 °C, monoterpenes and sesquiterpenes were emitted in lower quantities. This suggests a possible involvement of terpenoid modulation in the grapevine heat shock response. Also, 6-methyl-5-hepten-2-one could be an interesting compound, as genotype 66 increases its emission when subjected to heat shock. Information in the literature regarding this compound is very limited. However, it has been correlated with grapevine response to external stimuli, such as water deficit,^20^ and associated with leaf senescence in tobacco. Its concentration, in fact, has been reported to increase significantly during leaf yellowing.^43^ Therefore, 6-methyl-5-hepten-2-one could be a marker for leaf damage also related to heat stress.

#### Metabolomic Analysis

The PCA of metabolomic data obtained from UHPLC-ESI-HRMS analysis shows the separation between control (blue, Figure 6a) and stress samples (red) of CS in PC 2, while a partial separation between control and stress samples of the other genotype is visible in components 2 and 3 (Figure 6b).

**Figure 6:**
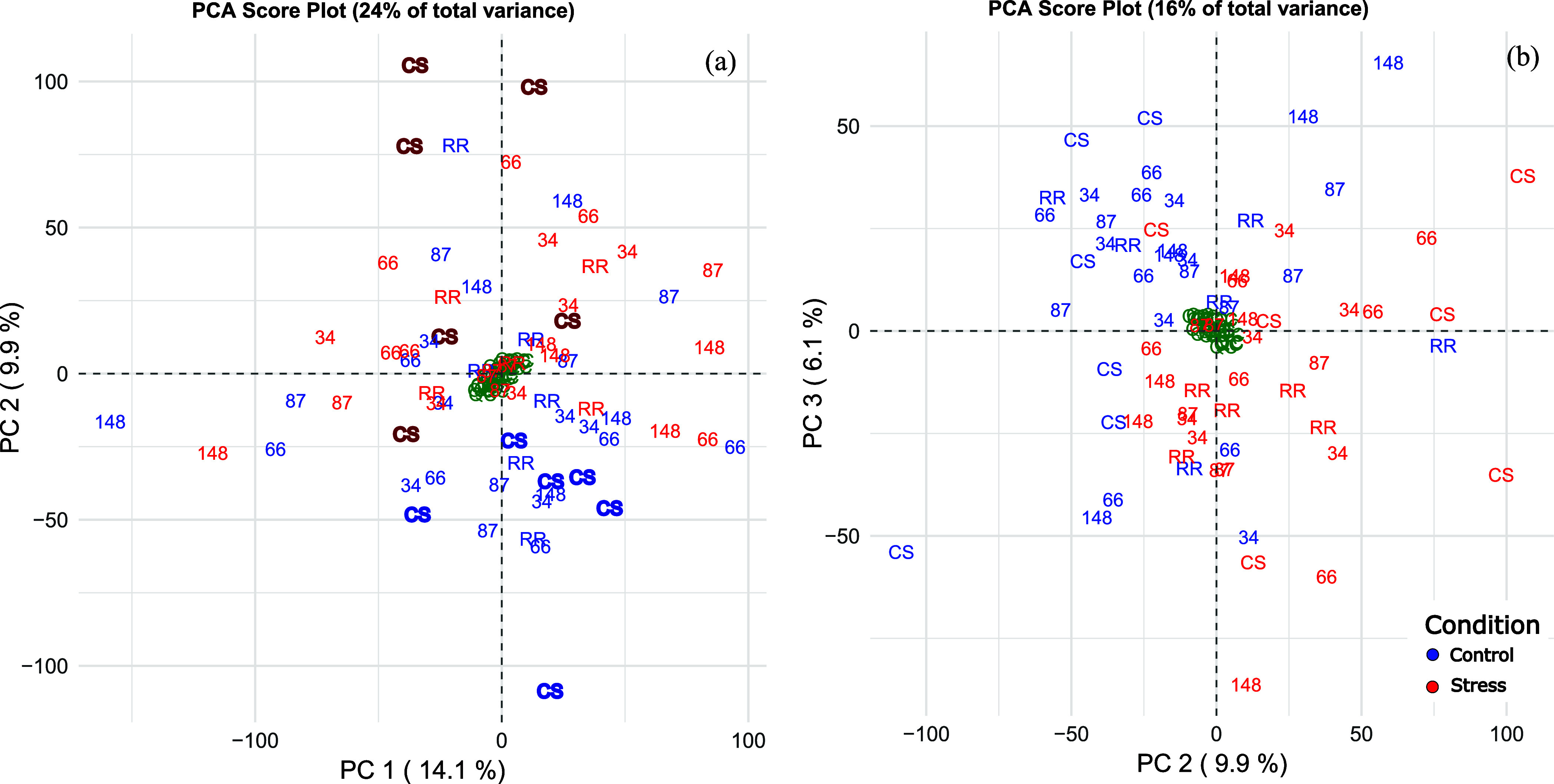
Score plots of components 1 and 2 (a) and components 2 and 3 (b) of the principal component analysis for metabolomic analysis (both positive and negative ionization modes) of 6 biological replicates of CS, RR, and genotypes 34, 66, 87, and 148 at control (blue) and after the application of a 43 °C heat shock (red). QC samples are reported in green. Each feature was normalized for QC, IS, and for the mean of the genotype to remove varietal contributions.

For each genotype, a *t* test between control and stress feature values was performed and significant features (*P* < 0.05) were highlighted both in negative and positive ionization modes. The frequency distribution of *P* values was also checked to exclude the possibility of false positives, and for each genotype and parental lines, there was an enrichment in features significantly altered during heat shock.

Among *m*/*z* values detected with MS-DIAL, 4185 features in positive and 8057 in negative ionization mode, only features with both *P* < 0.05 and effect size > |0.5| were considered for further investigation.

Cabernet Sauvignon had the highest number of features altered during stress among all the analyzed genotypes, followed by genotypes 66 and 148, while RR had the lowest number of altered features (Figure 7).

**Figure 7:**
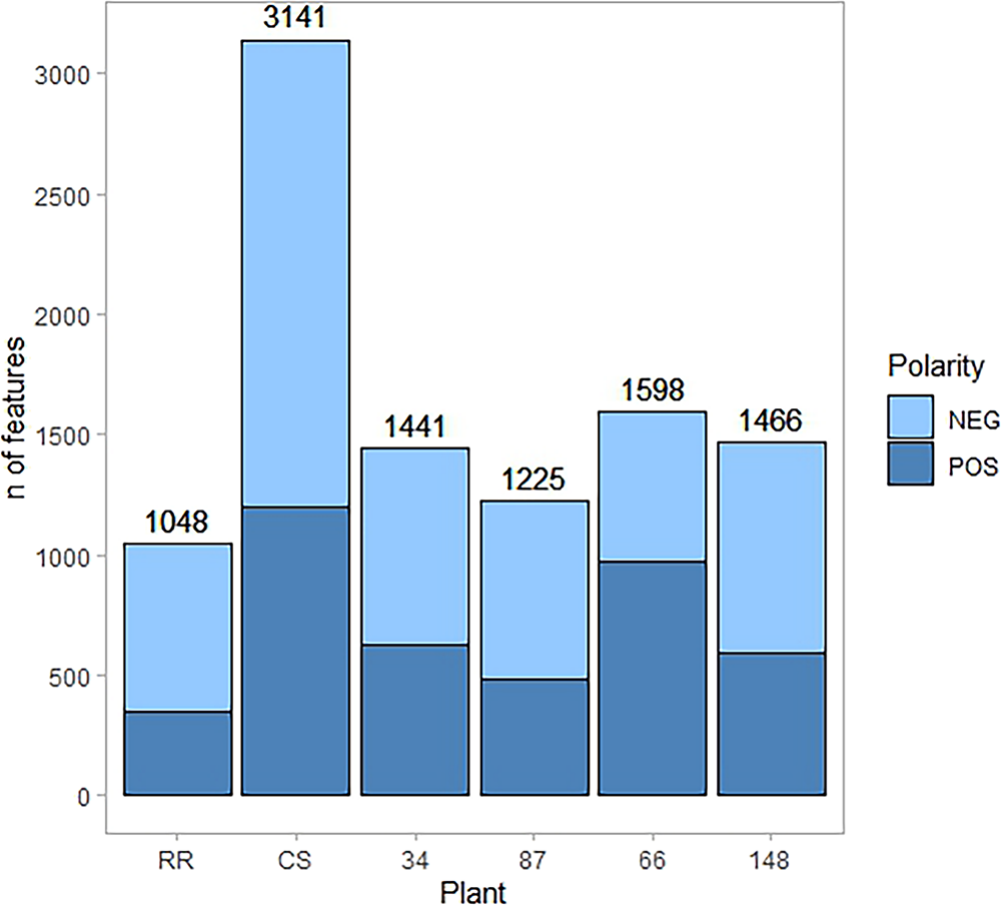
Number of features altered during stress, with *P* < 0.05 and effect size > |0.5|, for all the genotypes analyzed in this study, in positive and negative ionization modes.

Understanding the metabolic behavior of progeny genotypes and parental lines is complex. At 43 °C, in fact, reduction in F_v_/F_m_ was for every genotype around 15–20%, suggesting that all of them were stressed, but then, only genotype 66 never recovered, supporting the fact that genotype 66 could be considered susceptible. However, genotype 148 has a number of altered features that are similar to genotype 34, confirming its unique behavior, which is not in line with the prediction. In the conditions applied in this experiment, CS appears to be more responsive to heat shock application. Cabernet Sauvignon, in fact, exhibits a greater modulation of metabolites compared to RR, despite having a similar physiological response in terms of a decrease in the maximum quantum yield of PSII.

Among the features significantly altered with stress and with a big effect size, only a minor part (∼6%) was properly identified. For these features, Gene Ontology (GO) analysis was performed. Since the information given by unknown compounds was not considered and not all compounds within metabolic pathways are detectable with a single workflow, this analysis is not conclusive but can provide some indications on possible biological processes involved in the heat shock response regulation (Table 3).

**Table 3:**
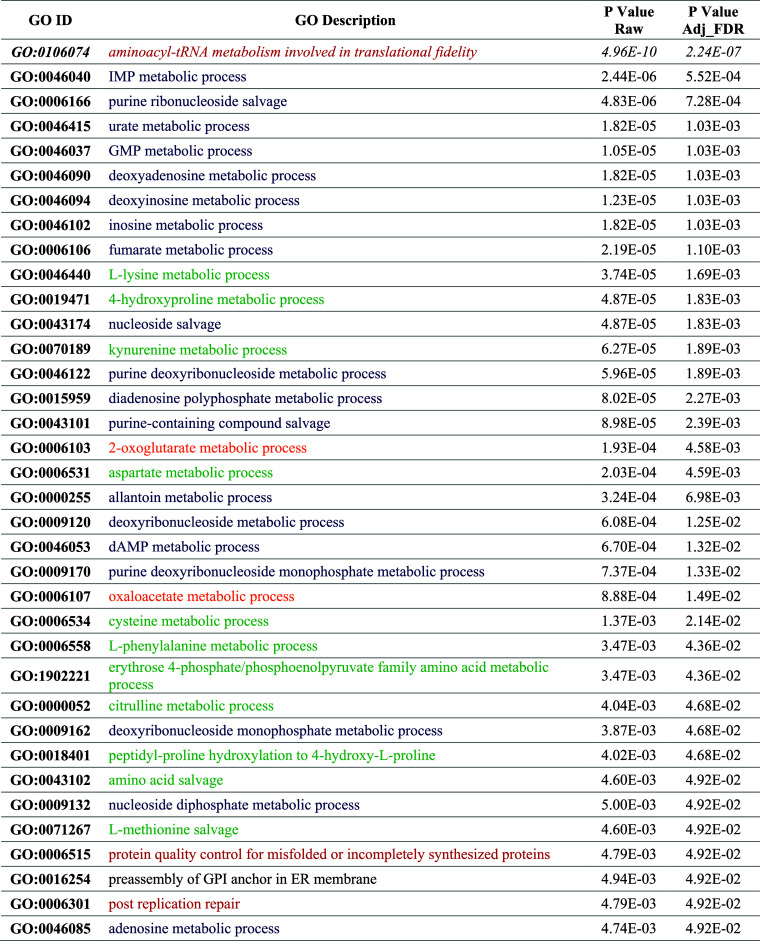
Gene Ontology ID and Description of Biological Processes Involved in Heat Shock Regulation^a^

a Results’ significance is reported as raw *P* value and *P* value adjusted for the false discovery rate (FDR). In magenta are terms related to protein synthesis and folding, in blue are terms related to purine and pyrimidine metabolism, nucleoside/nucleoside phosphate metabolic and salvage processes, in green are terms related to amino acids metabolic and salvage processes, and in orange are terms related to the TCA cycle.

The term with the lowest *P* value is related to aminoacyl-tRNA metabolism, which is involved in the correct translation of mRNA information during protein synthesis. This term has been related to heat stress in recent years in studies on different plants, such as wheat^44^ and *Sargassum fusiforme*,^14^ but no information is available regarding grapevine. Related terms are also the protein quality control for misfolded or incompletely synthesized proteins and post replication repair. Heat stress can increase cell stress; therefore, a proper mechanism of protein synthesis and folding and DNA repair is fundamental for plant thermotolerance. Evidence of the central role of these mechanisms in response to heat stress was also found in our previous work. In fact, several candidate genes related to heat shock proteins (HSPs), known to play a key role in preventing the misfolding and aggregation of proteins, emerged from the QTL analysis of the progeny.^25^ On the other hand, most of the GO terms are related to purine and pyrimidine metabolism, nucleoside/nucleoside phosphate metabolic and salvage processes (in blue in Table 3), and amino acid metabolic and salvage processes (in green). The purine salvage process has an essential function in several plant processes, and it is related not only to many physiological phenomena but also to the response to various stresses, such as wound or salt stress.^45^ Therefore, it is likely that these processes could also be influenced by heat stress. On the other hand, GO terms related to amino acids metabolism were identified also for wheat under heat stress,^44^ as well as terms related to tricarboxylic acid (TCA) cycle (reported in orange in Table 3).

Correlation tests between feature modulation and F_v_/F_m_ reduction highlighted 179 features in negative and 71 features in positive ionization mode correlated with F_v_/F_m_ alterations (with rho values in the range of ±0.3–0.6), but none of them was correctly identified by the software. Therefore, they were further investigated with MS-FINDER for the molecular formula and structure elucidation. All the features, with their spearman correlation value and tandem mass spectrum, are reported in Tables S6 and S7 in the Supporting Information. Among the features with the highest correlations with F_v_/F_m_ and tandem mass spectrum acquired, for *m*/*z* 773.2139 ([M + H]^+^, RT (min): 8.56, rho: 0.46), the formula with the highest score (3.87/5) and an error of −0.54 ppm was C_33_H_40_O_21_, corresponding to different possible structures, all belonging to the class of flavonoid-*O*-glycosides (in particular, a quercetin or kaempferol moiety with 3 attached sugar moieties). Other features putatively belonging to the class of phenolic glycosides were *m*/*z* 373.1503 ([M–H]^−^, RT (min): 10.16, rho: 0.40), *m*/*z* 681.2039 ([M–H]^−^, RT (min): 9.10, rho: 0.36) and *m*/*z* 343.0676 ([M–H]^−^, RT (min): 8.11, rho: 0.36). All these features were positively correlated with the decrease in F_v_/F_m_, therefore decreasing in higher magnitude during stress for genotypes which have a higher decrease in F_v_/F_m_. These findings are in line with what is reported in the literature. The phenylpropanoid pathway is known to be upregulated in plants in response to various abiotic stresses,^46^ with an increased concentration of phenolics, flavonoids, and anthocyanins. Therefore, a decreased amount of glycosides, which are the forms in which phenolic compounds accumulate in leaves, can be expected. Other interesting metabolites are amino acids and small peptides, which are in general accumulated significantly and with a big effect size during stress by all genotypes analyzed in this study (Figure 8). Accumulation of free amino acids has already been reported in many studies, summarized by Xu and Fu,^47^ and these metabolites are usually associated with the protein breakdown or produced as osmolytes in response to various abiotic stresses.^48^ Considering RR, which is the most tolerant among the genotypes studied in this experiment, glutamic acid, aspartic acid, methionine, and phenylalanine have an opposite behavior compared to genotype 66, which is the most “susceptible” in this study. Therefore, the modulation of glutamic acid, methionine, and phenylalanine could be related to differences in thermotolerance.

**Figure 8:**
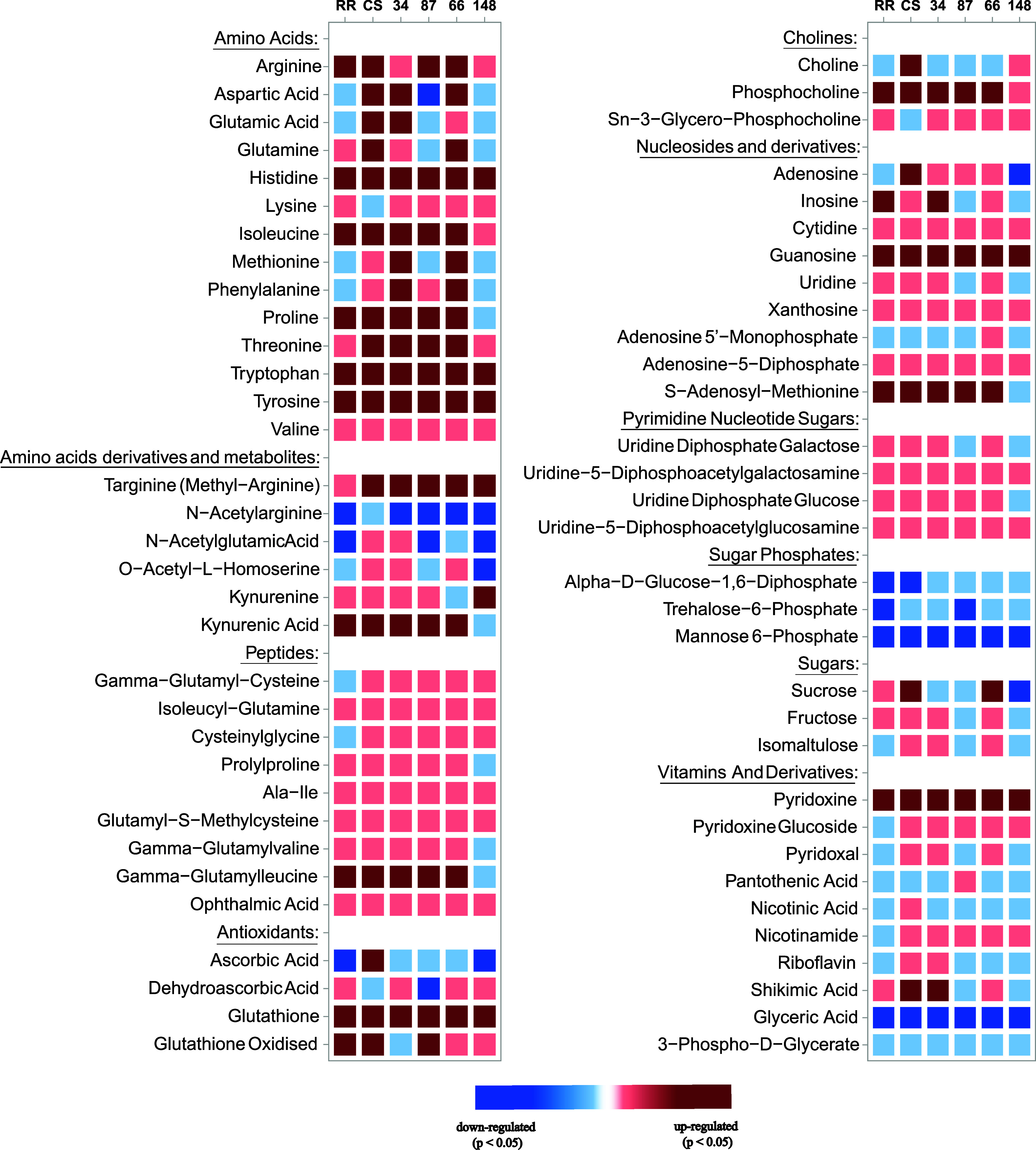
Heat shock-induced modulation of some interesting, identified metabolites for genotypes studied in this experiment (with genotypes 34 and 87 presumed tolerant and genotypes 66 and 148 presumed susceptible). Metabolite modulation was calculated as (HS – *C*)/*C* × 100, where HS is the area of the metabolite in the stressed sample (43 °C) and *C* is the area of the metabolite at control. In dark red and blue, metabolites are, respectively, upregulated and downregulated during stress (*P* < 0.05 and effect size > |0.5|); in light red and blue, metabolites have, respectively, a tendency to increase or decrease with heat shock but with *P* > 0.05.

Together with amino acids and peptides, nucleosides and nucleotide sugars tended to increase upon heat shock, especially guanosine, while sugar phosphates, glyceric acid, and derivatives were more abundant under control conditions (Figure 8). Conversely, shikimic acid levels increased more during stress in CS and genotype 66 compared to other genotypes. Additionally, pyridoxal, a form of vitamin B6, exhibited a unique response, while pyridoxine accumulated during stress across all analyzed genotypes. This is in line with findings from our previous QTL study. In fact, a gene coding for the positive regulator of vitamin B6 biosynthesis, pyridoxal 5′-phosphate synthase PDX1.2, was found in one of the conserved genomic regions highlighted in our previous work.^25^ Cabernet Sauvignon, when stressed at 43 °C, also increased the abundance of organic acids, such as caffeic acid, quinic acid, coumaric acid, and derivatives, together with bigger phenols such as epicatechin, kaempferol 3-glucuronide and quercetin, naringenin, and phlorethin glucosides. This behavior was not observed in other genotypes.

An interesting feature, which could be further investigated as a heat stress marker, is a compound with *m*/*z* 276.9885 ([M–H]^−^), which was significantly accumulated, and with a big effect size, only in genotype 66, the individual with the highest decrease in F_v_/F_m_ among the ones studied in this experiment, when heat shocked at 43 °C (Figure 9).

**Figure 9:**
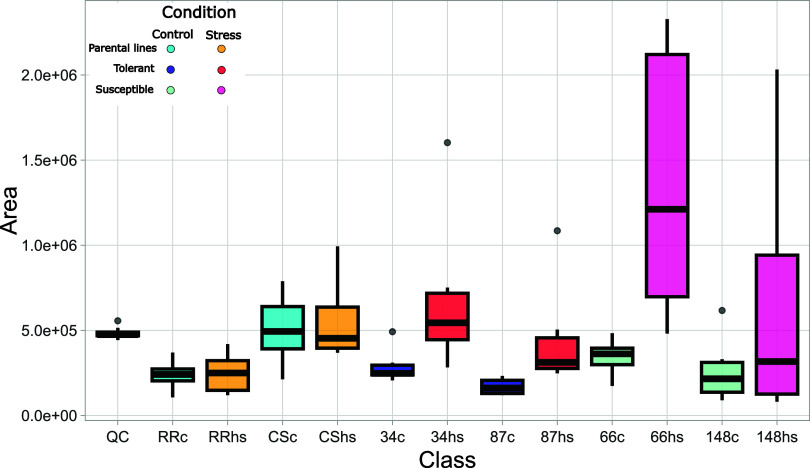
Boxplots of *m*/*z* 276.9885 peak areas, normalized for QC and internal standard, for different genotypes heat shocked at 43 °C. QC = quality control samples; RR = Rhine Riesling; CS = Cabernet Sauvignon. Progeny genotypes are reported with their codes. Control samples are coded with c, and stress samples are coded with hs. In blue (control) and red (stress), “tolerant” genotypes, while in aquamarine (control) and fuchsia (stress), “susceptible” genotypes. Results are reported as *N* = 6 biological replicates.

A tentative identification of the compound was performed with MS-FINDER software (no analytical standard was purchasable). The molecular formula with the highest score (3.6/5, error: 2 ppm) is C_5_H_12_O_9_P_2_, which corresponds to a single possible structure related to 2-*C*-methyl-d-erythritol 2,4-cyclodiphosphate (MEP-cPP, also referred to as MEcPP or MEcDP), with a score of 6.2/10. MEP-cPP is an intermediate compound in the MEP pathway for the synthesis of isoprenoids in plastids. Mass to charge ratios of other compounds belonging to the pathway were searched, and methyl erythritol phosphate (MEP) was tentatively identified with MS-FINDER; however, for this compound, there was no significant difference between control and stressed samples, suggesting a specific accumulation of MEP-cPP rather than a general alteration of the pathway. Moreover, in our QTL analysis for heat tolerance in grapevine, we found a gene coding for DXS (1-deoxy-d-xylulose-5-phosphate synthase), the first enzyme of the plastidial isoprenoid biosynthetic pathway.^25^ Our findings are supported by results reported in the literature. Studies performed in *Spinacia oleracea* and *Arabidopsis thaliana*, in fact, establish that the MEP pathway is not only responsible for isoprenoid biosynthesis but can also function as a stress sensor by modulating the levels of MEP-cPP, which functions as a retrograde signaling metabolite regulating the expression of stress-responsive genes when plants are subjected to high light, wound, or heat stress.^49,50^ Therefore, even if our results were not conclusive, MEP-cPP could also be involved in heat shock responses.

Results of metabolomic analysis performed on grapevines heat shocked under controlled conditions show that all genotypes studied are stressed at 43 °C, displaying an enrichment of features with a significant *P* value. The experiment performed does not highlight a clear trend distinguishing genotypes that could be more susceptible or tolerant when considering both physiological response and metabolites modulation. In fact, genotype 148 has a peculiar behavior compared to other genotypes analyzed and CS, despite having a F_v_/F_m_ reduction comparable with RR, is greatly influenced in its metabolome by the heat shock applied. Nonetheless, interesting putative markers of heat shock were identified.

#### Validation of Metabolomic Results in Field Conditions

To confirm the results obtained under controlled conditions, leaves of parental lines and of selected genotypes from the progeny were sampled to perform metabolomic analysis in two different seasons (2022 and 2023). Climatic conditions in the period between bud burst and harvest (April–September) were characterized in both seasons by moderate temperatures and approximately ∼600 mm of cumulative precipitation, well distributed over the growing season. Maximum temperatures during the growing seasons reached 36.6 °C in 2022 and 36.8 °C in 2023. Sampling was performed in the two seasons in July, during hot days. In 2022, the maximum ambient temperature reached during sampling was 35.4 °C, with Δ*T* = 17.9 °C between morning and afternoon values. On the other hand, in 2023, the maximum ambient temperature registered was 33.8 °C, with a temperature difference of 12 °C.^25^ Hence, in both seasons, ambient temperatures experienced by plants in the field were near optimal for grapevine growth. Nonetheless, the progeny is cultivated in Trentino–Alto Adige, a region in northern Italy characterized by cool climatic conditions; therefore, plants are likely to be better adapted to lower temperatures. Moreover, the temperatures recorded on the sampling days corresponded to the first hot days experienced by the growing grapevine shoots. Consequently, it is likely that no acclimation mechanisms were active during the course of the study.

When looking at the PCA of features extracted in both positive and negative ionization modes, PC 1 and 2 separate the control samples collected in the early morning (in blue) from the samples collected in the afternoon (red) in both seasons (in Figure 10 PCA of season 2023 is reported). Indeed, when performing *t* tests, significant features (*P* < 0.05) are highlighted. In Figure 10, the two groups are not perfectly separated, as stressed samples of genotypes 56, 161, and 188, for example, are in the same range of control samples. However, those genotypes were selected as presumed tolerant, based on their physiological behavior; therefore, a small difference between control and stressed samples is in line with our prediction.

**Figure 10:**
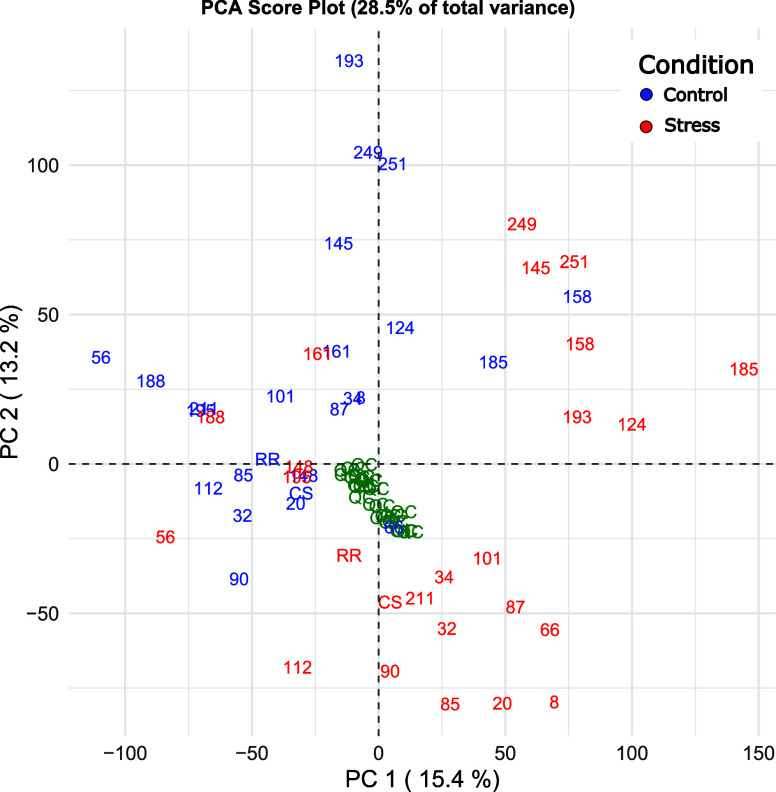
Score plot of components 1 and 2 of the principal component analysis for metabolomic analysis (both positive and negative ionization modes) of 2023 data normalized by QC and IS. Different genotypes are reported with their numerical code as a mean of three technical replicates, while parental lines are coded with CS (Cabernet Sauvignon) and RR (Rhine Riesling) and are reported as a mean of 5 biological replicates. Control samples are colored in blue, while stressed samples are colored in red.

Field behavior of parental lines confirms results obtained in controlled conditions. In both seasons, RR and CS experienced a decrease in F_v_/F_m_, which was between 1 and 6%, depending on the phenological stage at which the measure was taken. However, this difference in F_v_/F_m_ was not matched by a statistically significant modulation of their metabolome (*N* = 5 biological replicates), as reported also in controlled conditions for a heat shock at 40 °C.

On the contrary, *t* tests performed comparing tolerant and susceptible genotypes highlighted significant features, and the *P* value distribution plot showed a nonuniform distribution, suggesting the presence of an overall significant difference between the two groups. Among the modulated features, guanosine and *m*/*z* 276.9885 (hypothesized MEP-cPP), possible markers for heat stress, were found to be upregulated in susceptible genotypes compared to tolerant ones (Figures 11 and 12).

**Figure 11:**
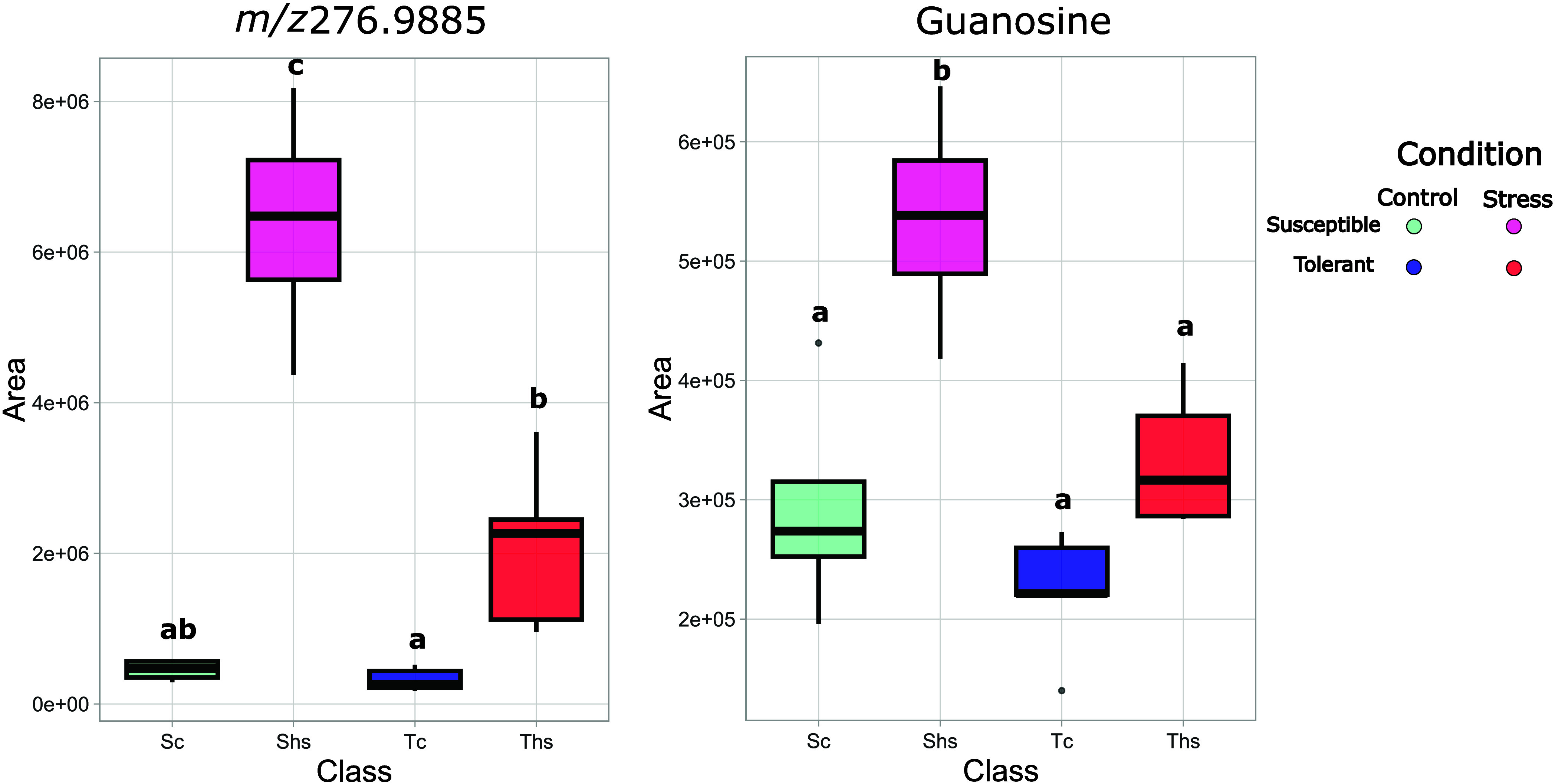
Boxplots of *m*/*z* 276.9885 (left) and guanosine (right) peak areas, normalized for QC and internal standard, for genotypes of the population sampled in 2022. Genotypes are grouped based on their physiological response in the field in “susceptible” (S) or “tolerant” (T). Control samples are coded with c and colored in shades of blue, while stress samples are coded with hs and colored in shades of red-fuchsia. Results are reported as *N* = 10 for the “susceptible” group and *N* = 13 for the “tolerant” group.

**Figure 12:**
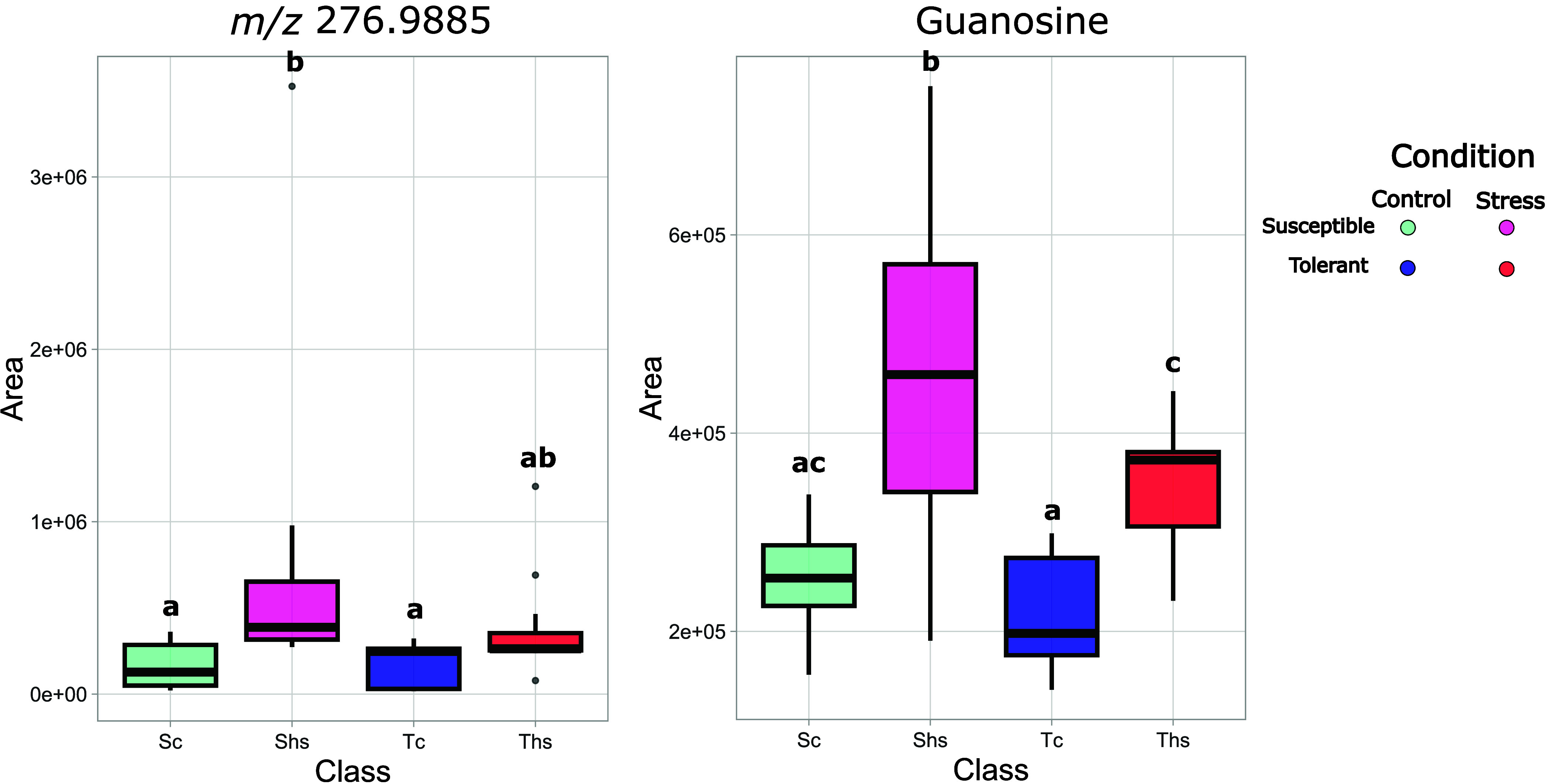
Boxplots of *m*/*z* 276.9885 (left) and guanosine (right) peak areas, normalized for QC and internal standard, for genotypes of the population sampled in 2023. Genotypes are grouped based on their physiological response in the field in “susceptible” (S) or “tolerant” (T). Control samples are coded with c and colored in shades of blue, while stress samples are coded with hs and colored in shades of red-fuchsia. Results are reported as *N* = 10 for the “susceptible” group and *N* = 13 for the “tolerant” group.

Other putative markers identified under controlled conditions did not show a statistically significant difference in their accumulation between susceptible and tolerant genotypes in the field. In fact, among amino acids, only serine and threonine were accumulated in the afternoon in all genotypes analyzed. Tyrosine, tryptophan, arginine, valine, and histidine, which were upregulated after heat shock application for all genotypes under study, did not highlight significant differences between morning and afternoon samples. The same trend was also visible for glutamic acid, phenylalanine, methionine, and shikimic acid, which were differentially accumulated in controlled conditions between susceptible and tolerant genotypes. This is probably due to the fact that plants in the field did not experience severe heat stress as temperatures were near optimal for grapevine growth in both years. Based on these results and on climatic conditions experienced by plants in the field, we can hypothesize that guanosine and the compound with *m*/*z* 276.9885, tentatively identified as methyl-d-erythritol 2,4-cyclodiphosphate (MEP-cPP), could be involved in an early response of grapevine to increased temperatures. Their accumulation, in fact, was higher in the 2022 season in which the ambient temperature was 1.5 °C higher compared to 2023, but with a difference between morning and afternoon temperatures 6 °C higher. Moreover, both compounds tend to accumulate in higher quantities in genotypes that have a higher impairment of F_v_/F_m_. Conversely, the other putative markers could be related to more severe stress, being differentially accumulated with higher temperatures, as they do not show significant modulation in the field but only under controlled conditions.

In this work, we integrated physiological, volatile, and metabolomic profiling to dissect heat shock stress response in grapevine through the study of two international varieties and their progeny.

Heat shock at 40 °C did not cause a significant change in plant physiology and metabolism, with only VOCs and the maximum quantum yield of PSII (Fv/Fm) mildly affected during the early stages of stress application, especially in Cabernet Sauvignon. Conversely, exposure to 43 °C elicited pronounced and genotype-dependent physiological and metabolic alterations across all genotypes. For five of the six tested genotypes, field-based thermotolerance rankings were reproduced in growth-chamber experiments. VOC analysis identified 6-methyl-5-hepten-2-one as a putative heat stress marker, rising exclusively in the most susceptible genotype. Across all genotypes, heat shock boosted the release of benzenoid volatiles (benzyl alcohol, 2-phenylethanol, and 2-phenylethyl acetate), increased several terpenoids, and suppressed monoterpene emission. Heat shock at 43 °C reconfigured grapevine primary metabolism: amino acids, peptides, nucleosides, and nucleotide sugars accumulated during stress, especially guanosine, while sugar phosphates, glyceric acid, and derivatives had higher levels at control. Finally, the nucleoside guanosine and the plastidial alarmone MEP-cPP emerged as heat-stress indicators, accumulating upon heat shock in heat-susceptible vines both under controlled conditions and in the field. Our integrated approach refines current knowledge of grapevine thermotolerance, delivers robust pipelines for multilevel phenotyping, and candidates guanosine and MEP-cPP as practical biomarkers to assist breeding and vineyard management under warming climates.

## Supplementary Material



## Data Availability

The data underlying this study are available in the published article and its Supporting Information. Metabolomic data are available in Metabolights repository: https://www.ebi.ac.uk/metabolights/MTBLS13195.

## Notes

Detailed parameters used in MS-DIAL software for volatile compounds analysis and metabolomic analysis (Table S1 and Table S2); VOC identified in the study (Table S3); physiological responses of genotypes in the field and in controlled conditions at 40 °C (Tables S4 and S5); genotypes’ physiological responses reported in percentage for the experiment at 43 °C (Figures S1 and S2); features from metabolomic analysis correlated with chlorophyll fluorescence decrease (Tables S6 and S7) (PDF)

## †

Department of Civil, Environmental and Architectural Engineering, University of Padova, Padova 35131, Italy
