# Supplementary material for: Decoding Heat-Shock Responses in Vitis vinifera L. by Metabolic, VOC, and Physiological Profiling: Toward the Identification of Volatile and Metabolic Biomarkers
Source: J Agric Food Chem. 2026 Feb 10;74(6):5796–813. doi: 10.1021/acs.jafc.5c14206 (PMC12921913; doi:10.1021/acs.jafc.5c14206)
Supplement: Supplementary file 1 [file jf5c14206_si_001.pdf]

SUPPLEMENTARY MATERIAL

Decoding heat-shock responses in *Vitis vinifera L.* by metabolic, VOC and physiological profiling: towards the identification of volatile and metabolic biomarkers

Silvia Pettenuzzo[1,2,3*], Marco Roverso[3], Michele Faralli[1], Pietro Franceschi[2], Laura Costantini[2], Emanuela Betta[2], Franco Biasioli[2], Sara Bogialli[3], Maria Stella Grando[1,2], Luca Cappellin[3]

[1]Center Agriculture Food and Environment (C3A), University of Trento, San Michele all'Adige, 38010, Italy

[2]Research and Innovation Centre, Fondazione Edmund Mach, San Michele all'Adige, 38010, Italy

[3]Department of Chemical Sciences, University of Padova, Padova, 35131, Italy

Table of contents

Table S1	*Parameters used in the MS-DIAL analysis of GC-MS data. Only parameters specifically modified for the analysis are reported, values suggested by software developers were kept for all the other parameters.*	2
Table S2	*Parameters used in the MS-DIAL analysis of LC-MS data. Only parameters specifically modified for the analysis are reported, values suggested by software developers were kept for all the other parameters.*	2
Table S3	*Volatile Organic Compounds identified with CLSA/GC-MS analysis of grapevine during heat shock in controlled conditions. For each compound identified, the retention time RT (min), Formula, Class, Ontology, Experimental Kovats Retention Index (RI) and literature RI are reported. Compounds identified with standards are reported in bold.*	3
Table S4	*Percentage values of chlorophyll fluorescence decrease ($\Delta F_v/F_m$ %, normalized for control values) at different sampling sessions (F= Flowering, Bp=Berry pea-size, Pre-V= Pre-Véraison, V=Véraison), for genotypes selected for metabolomic analysis in the field. Genotypes that tend to maintain their photosynthetic activity during hot days are highlighted in light blue while genotypes which decrease their photosynthetic activity are highlighted in orange. For each phenological stage, the maximum and the mean $\Delta F_v/F_m$ among the progeny are also reported. Differences in F_v/F_m > 10% are highlighted in bold, differences < 1% are reported with (-).*	4
Table S5	*Physiological response of genotypes when heat shocked for 3h at 40 °C. ΔFv/Fm and Δgs values were calculated as (C-X)/C *100, while ΔT leaf was calculated as (X-C), where C is the value at control and X is the value either at 40 °C or after 1-3 h of stress or after 1-24h of recovery. For each genotype at different time points is reported the mean value of N= 6 biological replicates. Colour codes are as follows: from green to red for increasing differences in Fv/Fm and increasing leaf temperatures; from green to yellow for increasing differences in leaf temperatures; from blue to red for gs variations, from stomata closure (blue) to stomata opening (red).*	5
Figure S1	*Rhine Riesling and Cabernet Sauvignon physiological response when heat shocked at 43 °C for 3 hours (N=6). Coloured dots show the trend of F_v/F_m and g_s measured at control (C, T= 25 °C), at the reach of 43 °C (HS), after 1-3 hours of heat shock (HS_1h, HS_2h, HS_3h), and at recovery after 1-24 hours since the end of the heat shock (R_1h, R_2h, R_ 3h, R_24h). For each condition, data were normalized values by the maximum value obtained at control.*	6

Figure S2	*Physiological response of genotypes 34, 87, 66, 148 when heat shocked at 43 °C for 3 hours (N=6). Coloured dots show the trend of Fv/Fm and gs measured at control (C, T= 25 °C), at the reach of 43 °C (HS), after 1-3 hours of heat shock (HS_1h, HS_2h, HS_3h), and at recovery after 1-24 hours since the end of the heat shock (R_1h, R_2h, R_ 3h, R_24h). For each condition, data were normalized values by the maximum value obtained at control.*	*7*
Table S6	*Features in negative ionization mode [M-H]- correlated with Fv/Fm (P < 0.05). Features are reported with their m/z, retention time (Rt min) and MS/MS spectrum when collected. Spearman correlations were calculated (rho).*	*8*
Table S7	*Features in positive ionization mode [M+H]+ correlated with Fv/Fm (P < 0.05). Features are reported with their m/z, retention time (Rt min) and MS/MS spectrum when collected. Spearman correlations were calculated (rho).*	*23*

Table S2 Parameters used in the MS-DIAL analysis of GC-MS data. Only parameters specifically modified for the analysis are reported, values suggested by software developers were kept for all the other parameters.

Peak detection	
Minimum peak height	10^5 (amplitude)
Smoothing method	Linear weighted moving average
Smoothing level	10 scan
Average peak width	20 scan
Identification	Use retention index (RI)
Index type	Alkanes (KOVATS)
m/z tolerance	0.5
EI similarity cut off	70%
Identification score cut off	70%
Alignment	Use retention time (min)
Retention time tolerance	0.1 min
EI similarity tolerance	70%
Retention time factor	0.5
EI similarity factor	0.5
Identification after alignment	v
Gap filling by compulsion	v

Table S3 Parameters used in the MS-DIAL analysis of LC-MS data. Only parameters specifically modified for the analysis are reported, values suggested by software developers were kept for all the other parameters.

Data Collection	
MS tolerance	0.01 Da
MS2 tolerance	0.025 Da
Retention time begin-end	0-18 min
MS range	100-1000
Peak Detection	
Minimum peak height	10^4 (amplitude)
Mass slice width	0.1 Da
Smoothing method	Linear weighted moving average
Smoothing level	25 scan
Minimum peak width	15 scan
Identification	
Retention time tolerance	100 min
Accurate mass tolerance	0.01 Da
Accurate MS^2 tolerance	0.05 Da
Identification score cut off	80%
Alignment	

Retention time tolerance	0.3 min
Mass tolerance	0.015 Da
Retention time factor	0.5
EI similarity factor	0.5
N% detected in at least one group	80%
Gap filling by compulsion	v

Table S3 Volatile Organic Compounds identified with CLSA/GC-MS analysis of grapevine during heat shock in controlled conditions. For each compound identified, the retention time RT (min), Formula, Class, Ontology, Experimental Kovats Retention Index (RI) and literature RI are reported. Compounds identified with standards are reported in bold.

Compound	RT (min)	Formula	Class	Ontology	Experimental RI (Kovats)	RI range literature
**Beta-Myrcene**	13.19	C10H16	Hydrocarbons	Monoterpenes	1171	1137-1173[a]
2-Heptanone	14.23	C7H14O	Ketone	Aliphatic compounds	1191	1178-1190[a]
**Gamma-Terpinene**	17.26	C10H16	Hydrocarbons	Monoterpenes	1251	1213-1261[a]
**Beta-Ocimene**	18.00	C10H16	Hydrocarbons	Monoterpenes	1266	1240-1270[a]
Hexyl Acetate	19.05	C8H16O2	Ester	Aliphatic compounds	1287	1270-1290[a]
3-hexen-1-ol acetate (Z)-	20.85	C8H14O2	Ester	Aliphatic compounds	1332	1310-1320[a]
6-methyl-5-hepten-2-one	21.43	C8H14O	Ketones	Aliphatic compounds	1348	1330-1340[a]
Hexanol	22.13	C6H14O	Alcohol	Aliphatic compounds	1367	1350-1380[a]
3-hexen-1-ol (E)	23.17	C6H12O	Alcohol	Aliphatic compounds	1395	1366-1394[a]
2-Nonanone	23.29	C9H18O	Ketone	Aliphatic compounds	1400	1385-1397[a]
**Cis Linalool Oxide**	24.92	C10H18O2	Oxides	Monoterpenes	1454	1430-1450[a]
Cis-3-Hexenyl Butyrate	25.53	C10H18O2	Ester	Aliphatic compounds	1474	1460-1480[a]
**Trans Linalool Oxide**	25.78	C10H18O2	Oxides	Monoterpenes	1482	1432-1490[a]
**Linalool**	27.96	C10H18O	Alcohol	Monoterpenes	1562	1500-1560[a]
**Beta-Caryophyllene**	29.09	C15H24	Hydrocarbons	Sesquiterpene	1605	1570-1632[a]
**Acetophenone**	30.63	C8H8O	Ketone	Benzenic compounds	1668	1607-1670[c]
Humulene	30.92	C15H24	Hydrocarbons	Sesquiterpene	1680	1632-1700[a]
**Germacrene D**	31.88	C15H24	Hydrocarbons	Sesquiterpene	1720	1674-1742[a]
**(e,z)-Alpha-Farnesene**	32.29	C15H24	Hydrocarbons	Sesquiterpene	1738	1692-1748[c]
**(e,e)-Alpha-Farnesene**	32.82	C15H24	Hydrocarbons	Sesquiterpene	1761	1720-1760[c]
2-phenyl-2-propanol	33.15	C9H12O	Alcohol	Benzenic compounds	1776	1759-1782[c]
**Methyl Salicylate**	33.57	C8H8O3	Ester	Benzenic compounds	1794	1780-1800[a]
**Nerol**	34.13	C10H18O	Alcohol	Monoterpenes	1820	1800-1820[a]
(z)-Isocitral	34.18	C10H16O	Hydrocarbons	Monoterpenes	1822	-
2-phenylethyl acetate	34.45	C10H12O2	Ester	Benzenic compounds	1830	1785-1839[c]
**Trans-Carveol**	34.52	C10H16O	Alcohol	Monoterpenes	1837	1800-1874[c]
Benzyl alcohol	35.73	C7H8O	Alcohol	Benzenic compounds	1893	1860-1890[a]
Cis-3-hexenyl benzoate	40.75	C13H16O2	Ester	Benzenic compounds	2143	2069-2155[c]

a NIST Chemistry WebBook (https://webbook.nist.gov) Kovats RI, polar column, temperature ramp
b http://www.pherobase.com/database/kovats/
c PubChem (https://pubchem.ncbi.nlm.nih.gov/, source: NIST Mass Spectrometry Data Center) Kovats RI for standard polar columns

Table S4 Percentage values of chlorophyll fluorescence decrease ($\Delta F_v/F_m$ %, normalized for control values) at different sampling sessions (F= Flowering, Bp=Berry pea-size, Pre-V= Pre-Véraison, V=Véraison), for genotypes selected for metabolomic analysis in the field. Genotypes that tend to maintain their photosynthetic activity during hot days are highlighted in light blue while genotypes which decrease their photosynthetic activity are highlighted in orange. For each phenological stage, the maximum and the mean $\Delta F_v/F_m$ among the progeny are also reported. Differences in F_v/F_m > 10% are highlighted in bold, differences < 1% are reported with (-).

	2021		2022				2023*	
	F	V	F	Bp	Pre-V	V	Bp	Pre-V
	ΔFv/Fm %	ΔFv/Fm %	ΔFv/Fm %	ΔFv/Fm %	ΔFv/Fm %	ΔFv/Fm %	ΔFv/Fm %	ΔFv/Fm %
20	3.6	**11.2**	4.3	3.2	3.1	4.6	2.6	3.4
56	4.7	4.5	0.6	2.5	1.5	-	0.9	0.2
90	**13.0**	5.5	2.8	3.1	3.8	5.2	2.9	2.1
101	9.7	7.4	1.0	0.9	1.5	4.0	2.7	3.6
112	1.3	-	2.5	3.3	2.6	-	2.4	3.3
124	-	-	3.7	0.7	3.5	0.6	3.3	4.2
145	0.3	0.9	4.2	0.9	0.8	-	0.9	1.5
161	1.0	-	1.8	1.2	2.8	1.2	2.0	1.6
188	0.8	-	1.3	1.4	4.1	1.8	2.4	1.7
249	1.8	-	2.1	0.2	0.9	-	1.4	1.9
8	4.0	9.1	6.4	2.9	**-**	3.4	8.2	**10.4**
32	-	1.6	4.6	4.3	3.6	6.1	**9.9**	**12.4**
85	4.8	2.3	**11.9**	4.8	1.5	0.7	8.9	**11.6**
158	8.2	2.3	**9.9**	5.5	6.1	7.2	**10.7**	**12.3**
185	-	2.0	**10.2**	**12.5**	**11.2**	**13.0**	6.8	5.5
193	**10.1**	-	**12.1**	4.6	3.0	1.8	**14.3**	7.7
195	2.4	-	8.8	9.1	5.9	**10.2**	9.2	6.1
211	0.1	0.7	7.3	5.7	**12.3**	**11.1**	**10.7**	8.1
251	7.6	3.7	**15.0**	-	5.0	3.0	9.6	**10.6**
Max	23.4	11.2	15.0	13.8	13.3	14.4	14.3	20.4
Mean	3.9	2.5	5.7	4.7	3.6	3.3	5.2	5.2

* Dataset *Flowering 2023* was not considered because temperatures were too low (Pettenuzzo et al. 2025)

*Table S5. Physiological response of genotypes when heat shocked for 3h at 40 °C. ΔFv/Fm and Δgs values were calculated as (C-X)/C *100, while ΔT leaf was calculated as (X-C), where C is the value at control and X is the value either at 40 °C or after 1-3 h of stress or after 1-24h of recovery. For each genotype at different time points is reported the mean value of N= 6 biological replicates. Colour codes are as follows: from green to red for increasing differences in Fv/Fm and increasing leaf temperatures; from green to yellow for increasing differences in leaf temperatures; from blue to red for gs variations, from stomata closure (blue) to stomata opening (red).*

		40°C	1h	2h	3h	R_1h	R_2h	R_3h	R_1gg
F_vF_m (% to control)	RR	7.0	6.5	5.8	3.6	0.9	1.4	1.2	3.4
	CS	4.4	6.0	3.7	0.9	2.0	1.3	1.8	0.6
	34	3.2	5.9	4.4	1.2	0.3	1.2	1.3	0.5
	87	4.1	5.8	4.6	1.5	0.7	0.9	1.0	0.3
	66	7.3	8.6	9.5	7.5	0.5	1.2	1.7	1.5
	148	5.1	6.4	3.8	1.3	0.4	0.4	0.8	0.2
g_s (% to control)	RR	30.9	6.2	-3.2	34.8	52.3	16.9	18.9	-10.6
	CS	6.3	-37.8	-27.9	-31.4	38.8	34.4	27.6	-26.1
	34	36.5	15.1	29.3	44.1	60.9	71.3	61.8	54.3
	87	32.5	45.8	57.6	35.2	36.9	54.2	21.6	6.5
	66	59.4	52.4	59.3	71.3	15.0	13.7	11.0	11.5
	148	-30.5	-52.5	-73.5	-18.6	12.3	28.6	31.3	-34.5
Tleaf	RR	29.5	29.9	30.7	30.1	26.9	26.4	26.3	25.9
	CS	30.1	30.7	30.8	30.7	26.9	26.6	26.8	24.6
	34	27.3	28.2	29.1	28.2	25.5	24.8	24.7	23.4
	87	27.5	28.3	29.2	28.3	24.5	24.3	23.5	23.2
	66	28.7	29.1	29.6	28.2	23.1	24.4	23.7	24.8
	148	30.5	31.3	30.8	31.6	28.4	27.3	27.4	24.5
ΔT leaf	RR	5.0	5.4	6.3	5.6	2.5	2.0	1.9	1.5
	CS	5.2	5.8	5.9	5.8	1.9	1.7	1.9	0.3
	34	3.0	3.9	4.8	3.9	1.2	0.5	0.4	-0.9
	87	3.0	3.8	4.7	3.8	0.0	-0.2	-1.0	-1.3
	66	5.4	5.9	6.4	5.4	-0.1	1.2	0.5	1.6
	148	5.9	6.7	6.2	7.0	3.8	2.7	2.8	-0.1

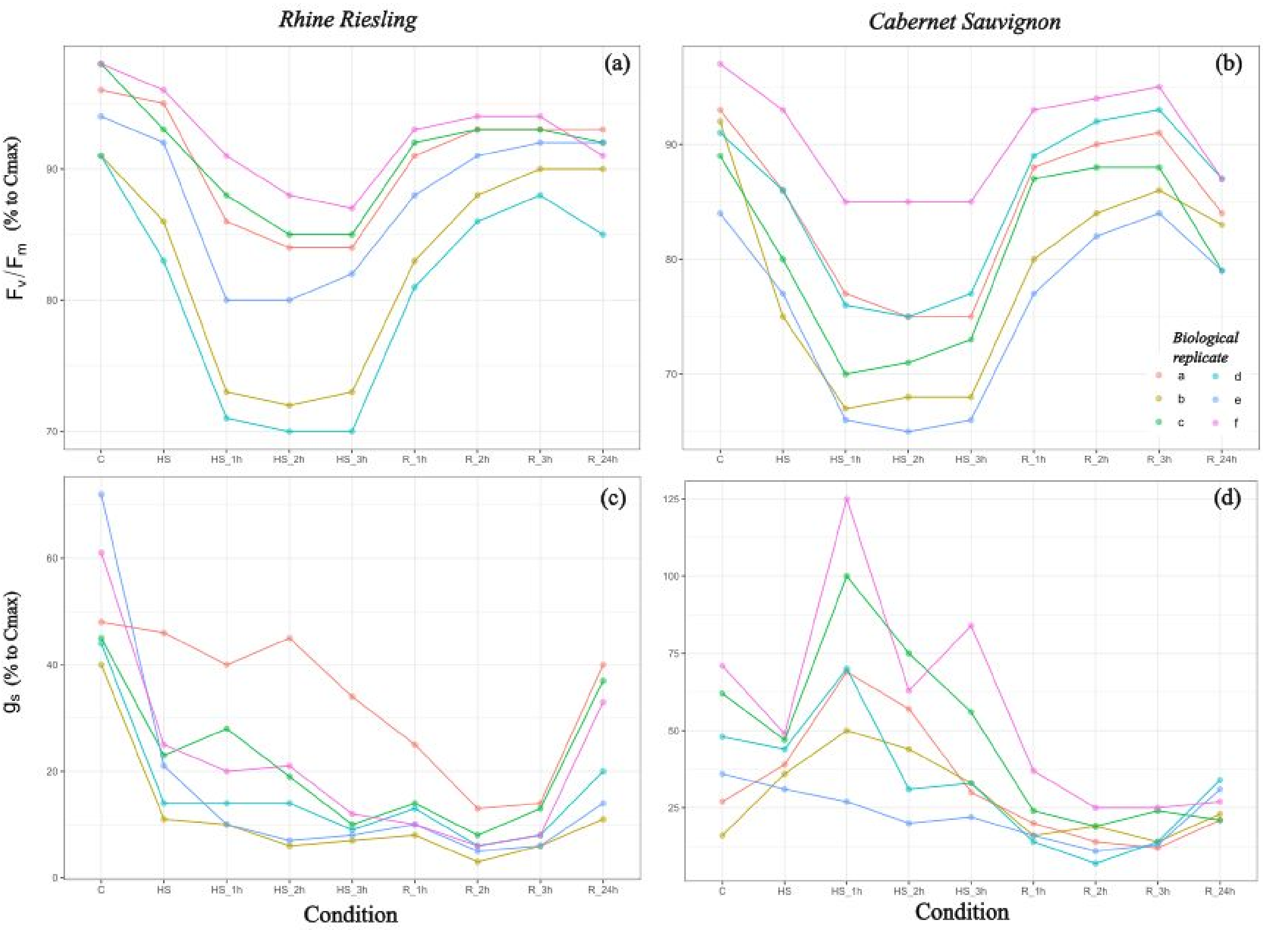

Figure S1. Rhine Riesling and Cabernet Sauvignon physiological response when heat shocked at 43 °C for 3 hours (N=6). Coloured dots show the trend of F_v/F_m and g_s measured at control (C, T= 25 °C), at the reach of 43 °C (HS), after 1-3 hours of heat shock (HS_1h, HS_2h, HS_3h), and at recovery after 1-24 hours since the end of the heat shock (R_1h, R_2h, R_ 3h, R_24h). For each condition, data were normalized values by the maximum value obtained at control.

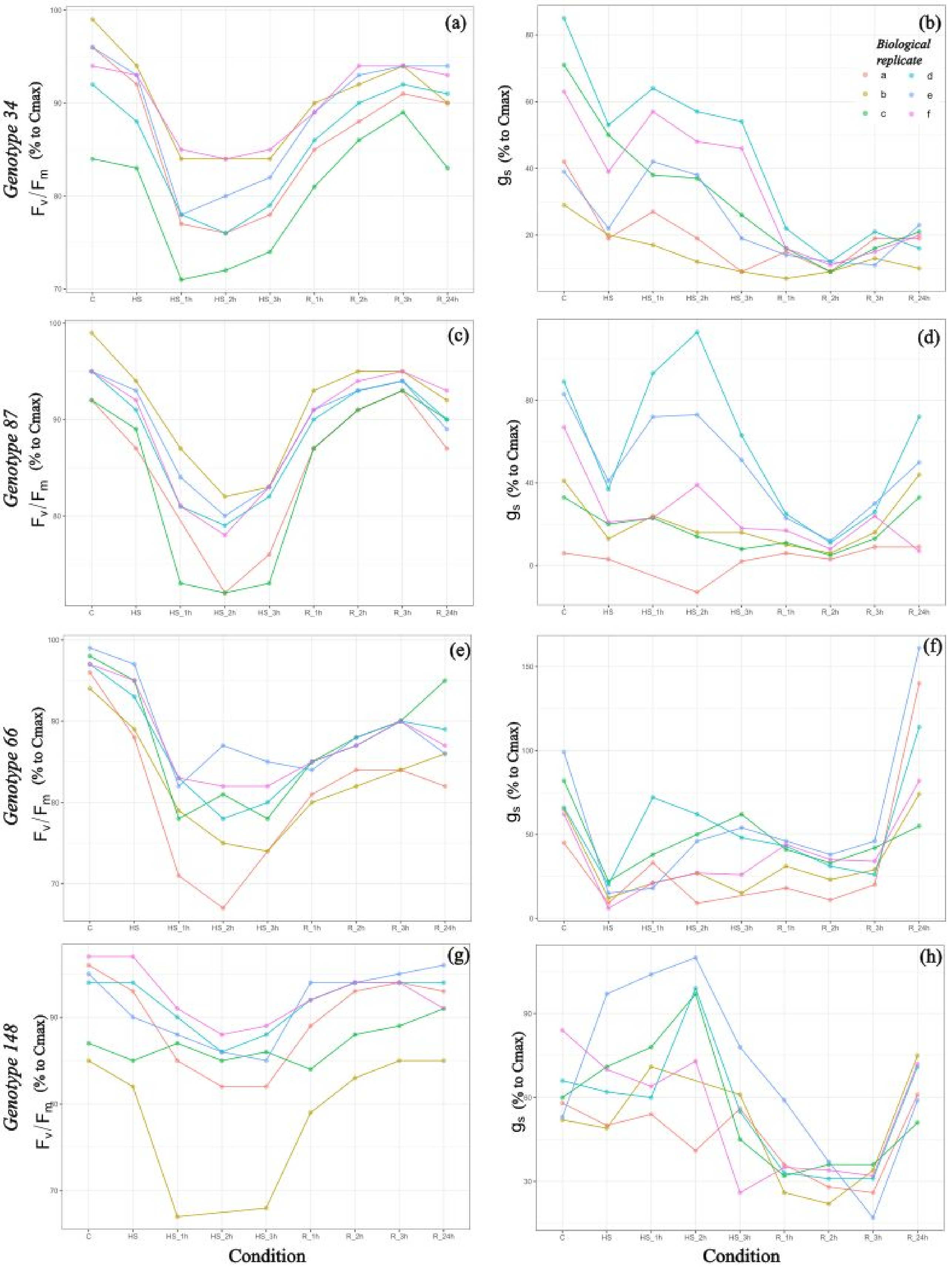

Physiological response of genotypes 34, 87, 66, 148 when heat shocked at 43 °C for 3 hours (N=6). Coloured dots show the trend of Fv/Fm and gs measured at control (C, T= 25 °C), at the reach of 43 °C (HS), after 1-3 hours of heat shock (HS_1h, HS_2h, HS_3h), and at recovery after 1-24 hours since the end of the heat shock (R_1h, R_2h, R_ 3h, R_24h). For each condition, data were normalized values by the maximum value obtained at control.

Table S6 Features in negative ionization mode [M-H]- correlated with Fv/Fm (P < 0.05). Features are reported with their m/z, retention time (Rt min) and MS/MS spectrum when collected. Spearman correlations were calculated (rho).

Rt (min)	*m/z*	*MS/MS spectrum*	*p.value*	*rho*
11.2	104.3575		0.0339	0.36
2.3	133.1693		0.0442	-0.35
1.8	166.9325		0.0344	-0.36
2.0	172.0615		0.0193	0.40
8.8	183.066	57.0346:1353 59.0138:12189 63.6623:1362 66.4332:1124 67.8908:1354 71.0136:3127 72.0216:4839 72.9931:1634 73.0297:1364 75.0085:2689 75.3111:1366 80.0266:1461 81.0710:4297 85.0293:1760 86.0010:35278 87.0040:2467 87.0087:12643 93.0791:1791 95.0503:16622 97.0660:3776 99.0815:15589 107.0503:17968 107.4904:1205 107.6947:1486 109.0294:1350 109.0658:18708 111.0816:38665 112.0167:1952 113.0612:2074 113.9959:193713 114.9991:5583 121.0661:4134 123.0456:9012 125.0609:36298 125.2568:1369 126.0640:2490 127.0764:38555 128.0795:1674 137.0608:16507 139.0766:5559 139.9926:1624 153.0561:5847 155.0714:50847 156.0745:4416 165.0559:12646 183.0663:36417 183.0817:2761 183.1026:2866	0.0194	0.40
9.4	187.0398	50.4539:933 53.8021:1015 77.4205:1244 79.8903:1530 89.3102:1370 102.1125:1414 114.5277:1491 122.0408:1225 125.0973:3099 131.0501:4004 158.0377:3016 159.0453:48205 165.4099:1426 187.0403:49606 187.0972:3568	0.0217	0.39
4.2	188.0931	52.0900:1104 56.9637:1536 69.0991:1479 71.0141:1360 84.5088:1154 85.9469:1495 90.9488:4152 97.9467:4690 98.9547:1799 99.0457:1582 99.9621:14197 100.0773:2811 100.9538:27339 101.9419:1676 113.0246:1968 113.9416:7357 114.0928:8339 118.0356:1334 122.1243:1379 129.9367:17940 130.9367:2826 141.9371:2871 142.0878:44742 142.9449:15744 143.0913:2442 143.9156:3050 143.9532:7804 144.9235:46196 144.9428:10970 146.0828:4010 157.9320:8635 175.9428:1731 186.9346:7435 187.0985:1622 188.0934:122031	0.0392	-0.36
8.8	211.0974		0.0185	0.40
10.8	212.02	50.0908:1125 53.4022:1109 56.6253:1166 56.7980:1097 59.8344:1347 68.0412:1433 80.0507:35078 90.3750:1326 107.6058:1385 124.0405:191759 125.0440:3363 168.0303:129311 169.0342:4053 211.1352:1739 212.0205:19598	0.0407	0.35
2.0	218.9818		0.0276	0.38
7.0	246.0982	51.0936:1013 53.1614:1040 58.4253:1292 62.1988:1328 63.9062:1201 71.0134:1778 72.9930:2013 73.3541:1352 76.1005:1325 103.5080:1385 109.0167:5931 116.9287:2422 130.0873:25413 140.1082:4289 151.4143:1372 155.9517:2382 155.9710:2023 166.0872:3049 184.0977:3657 210.0771:28504 228.0875:10234 246.0984:2390	0.0121	-0.43
1.9	257.0071		0.0019	0.51
11.6	265.1453	51.8761:1402 52.9574:1218 52.9653:1299 54.4295:1275 57.0346:12920 59.1272:1565 62.7085:1285 62.7296:1381 63.3922:1323 63.5283:1187 72.9884:1326 75.4941:1331 83.0504:7346 96.9603:2125 97.0659:18039 109.5425:1654 111.0452:47442 119.0864:2423 125.0609:20946 134.0757:1567 138.0685:4857 139.0764:3512 145.1023:9709 152.0844:2384 153.0921:102626 165.0918:12374 177.0558:4774 179.1078:6521 193.4675:1500 203.1248:2879 203.1442:55318 206.1303:1702 219.0510:1846 221.1346:2848 221.1548:35331 246.1435:7162 247.1331:1821 264.1548:9865 265.0346:1710 265.1458:61184	0.0264	0.38

Rt (min)	*m/z*	*MS/MS spectrum*	*p.value*	*rho*
3.0	273.0235	53.5251:1969 56.1254:1940 57.0348:2188 59.0139:5661 71.0139:17590 77.9134:2312 79.9575:10578 80.9168:4781 87.0087:8394 96.9601:1384580 99.3648:2267 113.0245:3111 114.9957:4987 115.0037:85041 115.5813:2228 128.0353:6502 129.3875:2804 136.1146:2534 143.0462:15139 175.0248:480822 214.3260:3036	0.0054	0.47
2.3	274.0259		0.0248	-0.38
10.1	281.1399		0.0445	0.35
1.8	281.8936		0.0255	-0.38
12.4	283.9959		0.0162	-0.41
8.8	285.0248	50.5680:1381 50.6799:1258 53.9003:1405 69.2346:1320 82.5375:1492 86.9411:1412 93.0805:2198 99.0092:2102 101.2962:1528 101.9287:1410 119.0502:176549 120.9964:575196 123.2591:1497 129.3400:1444 130.2228:1651 155.4605:1761 163.0401:48704 208.7799:1829 232.5501:1809 234.1964:1820 238.4603:1783 284.1334:4943 285.0400:25807 285.1348:2754	0.0382	-0.36
2.2	288.1203		0.0277	-0.38
9.5	299.0607	59.0137:1776 60.7636:1689 63.9626:1277 72.9931:2294 73.9831:3603 74.001:8765 74.9909:11169 91.9939:8433 99.0084:2867 101.0244:2580 117.9731:22805 129.1129:1733 133.6575:1705 135.9836:197199 136.9916:27025 137.0245:9668 139.5240:1581 142.0637:1704 165.0562:2295 166.0585:2977 181.0324:2718 254.1587:3399 256.1745:4398 298.1478:9364 299.0810:2073 299.1140:4019 299.1516:4782	0.0207	0.40
3.0	299.8988		0.0387	-0.36
2.0	300.9459		0.0037	0.48
11.4	329.0517		0.0080	0.45
11.8	331.0093	54.0101:1712 74.0247:11588 105.3747:2172 108.0039:4554 114.6366:2058 114.7645:1873 148.7095:2063 151.9939:11255 203.5523:2218 238.1649:3873 243.0254:4235 243.0486:57605 243.1408:2960 256.1738:19580 259.0247:9162 280.1351:2128 282.1198:3490 282.1531:12368 284.1329:14389 287.0200:282038 288.0240:2310 298.1484:6625 298.1832:4152 300.1638:54511 302.2006:2619 321.1799:2360 331.0098:62805 331.0686:12227	0.0153	0.41
8.6	336.0951		0.0399	0.35
1.8	336.4333		0.0325	-0.37
1.8	338.8838		0.0079	-0.45
12.4	339.1064		0.0475	0.34
10.1	341.0886		0.0116	0.43
8.1	343.0676	59.0138:140835 68.9327:48409 71.6349:55175 87.0087:554398 103.0037:136159 112.9879:341868 130.9985:87254 133.4612:56656 135.0449:19957814 136.0484:436481 139.5710:65639 142.8630:55977 149.0088:16042416 150.0124:206350 179.0348:1456117 181.0504:1090291 311.0422:85784	0.0348	0.36
13.9	347.1863	59.1896:1306 62.6971:2146 69.0345:6104 73.2100:1368 78.9593:1755 92.1081:1516 92.4335:1438 93.0764:3228 117.4499:1468 129.8171:2050 233.1543:4161 300.1612:2431 301.7819:1654 303.1965:36426 304.2006:1763 347.1861:29232 347.2248:3194	0.0176	0.40

Rt (min)	*m/z*	*MS/MS spectrum*	*p.value*	*rho*
6.4	350.0652	50.4755:1344 51.4583:1353 71.0137:6699 72.1357:1482 73.0296:2343 100.9537:1673 101.0244:4644 101.2217:1640 102.3032:1587 102.9485:3024 113.0243:3763 115.9492:2451 116.9480:2808 117.9357:7010 118.9433:5982 140.1368:1584 144.9432:11481 146.9385:11036 156.5507:1929 157.9054:2334 159.0293:2277 159.9397:3121 160.9374:1765 161.0457:9674 162.9335:7352 172.8834:9918 190.9273:3585 192.4959:1660 203.9296:2489 216.8727:13909 218.8698:3256 231.8761:2650 241.5013:1676 259.8723:4359 260.8626:2005 266.1687:1930 321.871:1944	0.0319	-0.37
1.9	355.0888		0.0312	0.37
1.8	355.8606		0.0212	-0.39
9.0	360.5		0.0293	0.37
10.2	371.0988	51.6810:1582 56.4503:1548 59.0141:10658 66.4781:1617 71.0139:3532 72.4490:1663 80.9748:1774 87.0089:3905 89.0245:2880 89.5273:1538 91.0191:2880 107.0138:2206 115.8883:1919 135.0090:26893 137.6583:1758 149.0242:2175 163.0407:3171 166.0274:36920 167.0352:445444 184.9482:1924 209.0461:2790 212.4578:1805 221.0453:2392 264.7923:1729 300.0785:1759 311.0778:5066 314.1349:1850 329.0855:2250 337.8082:1708 341.2353:1982 371.0996:5256 371.1974:3883 371.2445:78732	0.0477	0.34
10.2	373.1503	53.5903:1452 58.0352:1395 59.0137:16270 60.2962:1399 69.9581:1495 71.0138:10859 83.0251:23039 84.0090:19509 85.0294:2275 86.4707:1679 89.0245:17477 93.0745:2055 101.0245:12108 109.0293:3836 113.0246:10252 119.0348:8207 125.0244:4360 137.0245:12639 137.0611:2418 149.0970:11940 151.0392:2139 166.4322:1886 167.0349:6294 167.1077:331123 168.0383:3612 168.1108:7188 175.0755:2274 179.0345:3058 179.0561:3343 191.1077:12764 193.0867:10814 205.0509:3570 211.0975:154259 212.1012:7553 235.0977:10526 245.0815:14857 253.1085:5824 289.0720:53634 290.0756:5125 327.0279:11563 343.2495:4009 372.2479:14849 373.1847:4460 373.2597:119848	0.0178	0.40
9.1	373.5259		0.0489	0.34
12.2	387.1438	57.2082:1458 59.0138:5643 69.8905:1376 71.0138:8840 79.7577:1739 87.0085:1928 89.0244:23711 95.4367:1864 101.0244:9792 109.0294:3094 113.0247:8455 119.0350:10433 123.0448:2061 125.0243:3458 137.0244:3251 143.0352:4453 149.0454:2572 161.0459:8708 179.0345:3697 179.0561:27999 179.0707:2429 203.0719:4356 205.0508:6957 225.0554:15333 245.0817:24281 289.0719:143973 299.1284:3013 318.1760:3256 340.1566:17955 341.1092:74243 341.1576:9311 343.1179:20050 343.1531:1531 369.1332:3129 387.0953:2793 387.1457:17851	0.0434	0.35
13.2	394.2365	60.4432:1655 61.9883:193346 62.1661:1591 78.9590:80887 116.9286:3004 141.9360:1754 158.9388:2440 160.7456:1750 161.9530:1793 205.9870:1689 219.4780:1657 261.0089:1775 280.5495:1911 293.0501:2189 298.1476:4590 394.2356:38870	0.0391	-0.36
1.8	399.8534		0.0208	-0.39

Rt (min)	*m/z*	*MS/MS spectrum*	*p.value*	*rho*
13.2	401.1607	53.7319:1565 56.2214:1426 60.9945:1689 61.9881:2190 67.2833:1407 72.2089:1867 74.5862:1605 85.1978:1520 97.6770:1768 108.4104:1616 109.0294:7253 116.7707:1645 123.0451:16137 151.0404:4912 163.0397:5129 184.2936:1969 193.0505:2215 201.0165:2109 237.1122:11583 241.0088:3455 249.1137:2645 277.1075:45366 291.1236:7713 318.1739:1847 385.8956:2096 401.1015:3652 401.1598:36504	0.0100	0.44
5.9	401.1819	50.5945:1539 52.1531:1500 52.6378:1550 54.9547:1648 57.0346:1568 59.0139:5078 67.1879:1522 71.0139:5135 87.0089:1773 89.0246:3966 91.5940:1550 98.2637:1692 99.2607:1753 101.0246:2591 113.0244:3824 119.3791:2050 151.0765:2889 153.0559:3163 162.3580:1695 162.7687:1684 163.0768:3346 165.0934:1756 203.1080:9567 212.6604:1632 221.1187:24275 250.8492:2063 268.4882:1570 367.0369:2411 375.9673:1711 401.1086:3595	0.0251	0.38
2.2	402.1257	56.8280:1493 59.0138:5662 63.4338:1585 72.9934:8668 78.9591:4024 87.0088:27541 88.2363:1982 89.4044:1790 93.0739:1858 93.4560:1714 96.9601:1966 103.0036:6399 105.0194:4240 111.0201:4125 130.9991:2276 149.0092:710470 158.9255:6993 175.3996:1743 191.0566:4009 245.2826:2368	0.0153	0.41
9.1	420.0154		0.0356	0.36
7.8	421.2444		0.0397	0.35
6.7	422.1591	53.8477:1379 58.0257:1482 61.9885:10089 75.1485:1653 80.3844:1672 81.2730:1644 88.9881:8513 93.0811:3454 101.2748:1729 111.2390:1697 128.0356:10234 143.0460:10460 145.0291:2474 148.2308:1745 154.0622:2870 160.0077:5617 177.0349:3230 179.0462:3273 180.0416:2094 188.6123:1722 199.0084:2313 210.0885:5853 254.0787:10928 258.1914:2016 259.1196:2314 261.0092:2148 272.0889:15464 288.0669:7044 300.0993:7302 306.0769:37074 353.0496:2202 366.9057:1826 421.983:4254	0.0446	0.35
1.9	425.1062		0.0033	0.49
1.8	427.9167		0.0091	-0.44
6.4	432.9925	54.7388:1473 59.0137:4588 63.0117:1482 71.0138:3757 86.8306:1795 89.0243:13808 96.9601:2699 99.0092:1941 101.0244:3629 112.5376:1726 116.9287:2999 119.0345:2255 125.0245:5493 131.0342:2019 149.0459:2201 179.0888:1705 188.6293:1793 191.0583:2132 226.9496:47712 233.0671:9176 239.7175:1745 241.4869:1853 242.9441:22103 254.9443:4267 284.9559:9635 293.0876:16892 356.9794:2252 384.9864:2506 385.9885:8872 386.9869:63615 387.1284:2952 388.0005:3967 389.0017:9373 404.9978:3975 433.2069:3977	0.0332	-0.37
8.2	438.1214		0.0247	0.38

Rt (min)	*m/z*	*MS/MS spectrum*	*p.value*	*rho*
8.1	441.9853	59.0142:3022 60.5302:1668 61.9887:2689 68.7083:1737 71.0139:5721 73.0296:1867 83.0141:1872 89.0244:3286 90.5157:1733 91.3207:1682 96.9603:6587 101.0244:10157 109.7511:1839 113.0248:3331 115.9403:2304 116.9288:2851 119.0361:2320 131.0349:4297 135.0454:8812 141.6863:1848 142.9438:5411 148.0410:1904 149.0094:4931 149.0461:2417 161.0459:6803 168.9964:2788 171.9468:2280 172.9539:4492 186.9342:4309 190.9806:2717 203.9882:23083 206.9760:3737 217.9687:2988 232.9921:4418 233.9462:9214 234.9707:3118 247.9784:11212 248.9861:2883 249.1351:45420 250.1390:7931 259.9796:4091 262.9654:23134 263.1503:22836 263.9732:6032 264.1542:4133 275.9732:115745 276.4471:1967 276.9780:6465 291.9681:2586 302.2538:1896 306.9563:7114 319.9627:13841 321.1556:2695 338.9820:7729 352.0233:2377 380.9562:8628 395.1932:61823 395.9786:18850 396.1967:16951 406.9760:2338 413.9912:4281	0.0248	0.38
9.7	443.0449	59.0138:3783 67.5285:1694 71.0138:5578 72.6685:1551 82.1073:1610 87.0087:3747 89.0247:2118 101.0245:6831 103.3678:1748 104.3483:1641 113.0244:4039 135.0456:1873 149.0093:44873 161.0455:5666 179.0351:26731 189.2257:1863 193.0877:1809 266.5519:1830 311.1517:9007 394.0018:3908 443.1905:5758	0.0142	0.42
11.0	443.2273		0.0392	0.36
8.2	444.2152	51.9622:1468 59.0139:2679 68.1162:1760 74.3375:1690 101.0247:2272 103.0127:1925 116.9286:2248 117.9286:2658 123.0382:2028 123.046:8077 146.1579:1907 149.0248:13089 260.9673:2171 261.9800:4505 275.9712:2523 277.9783:3530 294.1832:122552 305.9698:3044 319.9941:3358 320.1626:3884 320.6079:1868 349.0953:2114 397.2000:3852 399.0099:2302 444.2153:542790	0.0084	0.44
3.0	445.1946	52.4211:1759 61.2118:1672 69.4525:1532 71.0138:2656 84.7741:1769 89.0243:2360 96.9598:4192 101.0238:2966 113.0243:2175 161.0442:2292 182.7627:1703 220.9761:4736 223.5681:1786 246.7390:1877 309.1189:2849 398.1772:2131 399.1840:3636 401.7510:2041	0.0339	-0.36
10.2	446.12		0.0017	-0.52
5.9	447.1881	54.3469:1643 59.0138:5259 71.0137:8094 73.0955:1719 73.9191:1600 85.0292:1983 89.0245:9964 96.9600:3232 96.9696:2871 101.0242:5257 113.0241:5438 119.0347:3539 134.2956:1834 135.0456:2014 158.9385:2241 161.0458:6160 179.0563:2772 203.1074:9552 221.1177:34826 326.9765:1852 341.7644:2237 351.6625:1956	0.0203	0.40
2.6	447.9588		0.0400	-0.35
13.1	450.2705		0.0391	-0.36
2.9	452.065		0.0130	-0.42
4.5	459.1566		0.0215	-0.39
2.1	462.9981		0.0208	0.40
11.4	465.0222		0.0052	-0.47
4.3	468.0203		0.0402	-0.35
10.3	469.0043		0.0478	-0.34
12.2	469.1899		0.0198	0.40
5.9	473.0253		0.0345	-0.36

Rt (min)	*m/z*	*MS/MS spectrum*	*p.value*	*rho*
2.1	480.8885	61.8105:2409 62.7102:2386 70.6276:2682 72.9931:2357 75.6905:2586 78.1102:2498 78.9597:19973 87.0085:3126 87.8866:2669 96.9602:5084 96.9696:28070 97.4678:2736 98.4206:3103 135.0299:6102 149.0091:429701 150.0129:8836 150.7660:2497 165.0414:3795 174.7383:2963 184.9855:4804 192.9215:3795 194.9466:11056 208.9184:3895 210.9310:4156 214.8811:13630 231.7781:2875 232.8949:55534 237.8972:7692 240.8970:3751 241.7581:2531 246.9774:9604 247.9812:6840 250.9044:6037 252.9435:2962 258.9089:23136 266.9223:150480 267.9261:4248 274.9040:4964 276.8390:7339 284.9328:1227662 285.9362:24338 292.9149:22256 294.8487:50616 296.8816:11841 300.8825:4076 302.8993:15673 306.9985:8587 312.8590:192884 314.8942:3525 328.8767:15304 330.8704:20676 346.8890:41032 346.9340:3590 346.9936:5685 364.8990:252046 365.0036:12715 365.7818:3532 382.9089:97720 382.9586:4641 383.0123:27304	0.0150	0.41
13.2	485.2397	57.2372:1437 61.6074:1563 61.9884:1552 65.299:1786 90.8731:1837 109.7014:2073 125.0883:2777 125.0970:39680 147.0427:1818 177.6947:1998 187.0973:4223 258.3271:2073 315.1444:58852 316.1484:2512 363.6865:1897 370.0890:2267 389.0513:5500 485.2383:58880	0.0450	-0.35
8.0	487.1115	50.5300:1689 55.9893:1592 59.014:21095 61.0930:1884 64.8188:2066 70.2250:2089 71.0137:6344 72.9930:2245 73.7455:1746 78.9590:2408 84.9838:1959 87.0088:22346 89.0244:7561 95.4033:1769 96.0094:2091 96.9604:11540 96.9697:17394 98.9557:3958 100.9339:1969 101.0248:13384 102.7721:1987 103.0038:12765 112.9883:45190 113.0245:7111 116.9292:2010 118.4635:2303 119.0349:3567 119.0503:5621 135.0455:2981 137.6161:2046 139.0767:64911 140.3018:2203 141.0558:2816 145.0145:18110 149.0093:3233 149.0609:6904 151.0402:6652 158.9383:4915 161.0455:7488 163.0403:59150 168.0796:12253 169.0139:2408 169.0875:9569 170.2404:2241 171.9469:4431 172.9548:28898 173.9584:4573 178.0268:3492 179.0343:2800 187.1132:2730 193.0508:340845 194.0544:15846 205.1234:16169 217.1234:2949 229.1240:2964 235.0614:4034 235.1344:8570 245.0815:3295 249.1139:8013 261.1149:2448 263.0673:2830 263.0923:13352 273.1130:7456 276.9663:4926 279.1241:170397 280.1275:10164 289.0383:3603 291.1249:2996 303.1237:15129 307.1768:3783 308.1803:10863 309.1838:2201 316.5821:2127 321.0675:2214 321.1348:9201 325.0960:3434 331.0682:4649 335.0784:2710 335.4708:2257 335.5094:2380 355.1038:13610 383.9794:4230 425.1462:8907 426.1466:2244 440.1132:12506 440.2213:12080 441.1205:60531 441.2225:6116 442.1328:17149 443.1337:5408 459.1323:5696 486.1162:13307 487.1296:4899	0.0460	-0.34
9.4	488.0252		0.0363	0.36
8.2	489.0708		0.0456	0.35
9.4	490.2035		0.0454	0.35
7.5	496.123	59.0138:5229 62.83:1504 68.9558:1585 71.0137:6569 73.0293:2058 75.6674:1669 76.4923:1655 89.0244:5074 101.0244:10835 112.9857:4818 113.0241:2879 117.1441:1654 119.0346:2296 125.0247:3286 161.0454:18024 163.0611:2683 215.4459:1734 247.0829:3798 304.0591:15867 315.0728:2421 392.1122:2147 431.9937:15741 432.9988:3016 450.0013:16109 450.1180:9085	0.0270	-0.38
9.5	500.1405		0.0428	0.35
3.2	502.1157		0.0452	0.35

Rt (min)	*m/z*	*MS/MS spectrum*	*p.value*	*rho*
6.0	503.1055	53.8593:1600 54.8823:1464 60.2429:1647 61.4196:1578 63.5247:1708 78.9591:4397 80.4401:1662 87.0087:3361 88.9882:2816 96.9600:59162 96.9695:44681 105.9251:2015 135.0452:9171 137.2991:2001 146.9387:3424 149.0090:91587 153.0555:4441 173.0453:3902 173.9667:2051 179.0351:4070 190.9287:5413 192.1504:2728 197.0454:6236 218.8690:2983 262.8586:5647 268.2928:1810 291.0180:2605 341.0882:4994 353.0876:2646 371.0993:4203 456.1132:10471 457.1138:3580 460.1414:3206	0.0347	0.36
10.6	503.2506		0.0454	0.35
3.1	506.0656	56.205:1516 78.9593:2176 96.9604:2141 99.0913:1614 108.8146:1809 134.1809:1968 145.9307:3261 157.8785:1844 158.9256:5716 234.9945:33291 281.0001:56782 386.8062:2319 408.0139:2898 414.8024:2994	0.0265	-0.38
10.6	506.1113	56.5797:1789 59.0111:3024 59.0139:52445 63.5968:1807 67.9399:1746 68.0255:175277 71.0141:20923 73.0260:2359 73.0296:28126 75.6298:1710 85.0293:5454 87.0089:2318 87.0452:2338 89.0246:45938 101.0246:64046 102.0278:4605 103.0403:33675 104.0433:2874 113.0245:5915 115.0403:5146 116.3272:2434 119.0352:27094 125.0244:3471 127.0406:2734 131.0348:6795 143.0350:13307 145.0507:5683 149.0088:4938 149.0455:8709 161.0456:4081 163.0614:77094 164.0646:7338 165.0927:5581 173.0453:3128 191.0567:3207 194.8299:2212 205.0719:44388 206.0755:3488 214.0906:1956 229.1155:2032 240.0204:2196 248.5686:2129 285.1133:3416 293.0889:2185 329.1397:23611 330.1435:7281 335.4466:2192 335.4891:2182 343.1216:3897 357.1974:2083 367.3695:2082 373.0963:2950 459.2176:3147 461.2371:6884	0.0281	-0.38
1.8	506.8774		0.0494	-0.34
10.6	508.1085	53.0165:1718 59.0111:2222 59.0139:35903 61.9883:2631 68.0255:182110 69.0287:4356 71.0139:22801 72.0058:1860 73.0296:17228 81.3105:1941 83.0137:2130 83.0251:4334 84.0091:4841 85.0295:5235 87.0089:2205 89.0245:44992 91.1104:1791 93.0741:2220 101.0178:3079 101.0245:62637 102.0278:3163 102.5925:1931 103.0401:22106 113.0245:10008 113.9261:1746 115.0400:3656 116.9283:5067 119.0350:30996 119.4958:1738 120.0386:2493 125.0244:4742 131.035:7207 135.0452:7655 143.0347:10282 145.0507:5927 149.0094:4159 149.0458:5650 161.0459:7838 163.0613:60590 164.0647:7531 167.1079:6527 179.0349:7708 179.0565:9343 205.0719:31861 206.0754:6490 265.0938:4843 301.0759:2174 315.0883:3709 315.1818:8656 316.1824:2469 327.1264:4232 337.2326:1933 377.0883:5292 461.2397:20758 462.2427:7391	0.0165	-0.41
11.2	511.1132		0.0219	-0.39
11.0	513.2344		0.0061	0.46
2.8	514.1793		0.0440	-0.35
1.8	514.8326		0.0199	-0.40

Rt (min)	*m/z*	*MS/MS spectrum*	*p.value*	*rho*
9.7	519.9783	56.5159:1560 56.7668:1684 59.2835:1663 59.9864:1591 64.4986:1790 65.4450:1805 87.0088:4320 95.2808:2092 133.0295:3143 134.0375:3396 135.0452:2915 149.0091:19247 161.0253:2842 170.4687:2362 177.0193:4114 178.0271:4407 179.0353:20368 226.6786:2041 227.9368:7680 232.9549:2745 247.9779:13462 248.9820:2227 250.9656:3586 274.9646:4026 276.4581:2849 285.8482:2108 291.9678:13015 292.9746:3917 298.4545:5700 298.9553:4805 306.9555:12189 307.9597:2400 319.9629:51115 320.9669:4989 327.9615:2289 348.9649:2203 351.0976:8342 352.0997:4730 363.9522:5699 364.9624:2383 374.8892:5128 384.1196:2534 391.8938:4714 395.0871:12323 396.0885:8068	0.0277	-0.38
9.3	520.1674		0.0065	0.46
12.2	531.316	57.9231:1855 59.0136:4200 71.0136:7073 87.1811:1711 89.0241:5787 96.9603:10880 96.9692:2169 100.4102:2180 101.0241:8419 109.0296:2339 111.9815:1789 113.0242:3681 119.0346:2926 125.0241:4682 135.0455:1955 161.0245:2713 161.0451:8498 199.0431:9599 199.0594:2155 210.1744:2440 211.2665:2085 237.1130:3359 243.1242:3735 271.0606:11654 300.0269:4230 301.0342:19156 323.2613:1979 338.4586:1883 377.8001:2169 433.1095:3045 440.3262:2108 463.0883:10784 485.2405:2751 485.3102:11302	0.0172	0.41
9.9	533.1308	53.6313:1913 59.0142:3152 60.9930:2524 66.6335:1852 79.6425:1723 87.0089:6716 89.0248:2680 91.4713:2233 93.0348:18060 93.0816:5062 96.4047:2147 101.0245:3266 109.0295:3101 111.0454:5372 111.2904:1917 135.0456:10635 149.0611:4585 155.0353:5394 161.0248:10398 161.0614:2855 173.0458:50693 175.0401:5173 179.0195:3126 179.0354:61986 191.0565:426304 192.0606:10585 241.0535:4989 243.0665:4567 255.0673:5484 271.0984:35762 272.0997:2555 287.0921:3744 289.0574:6659 297.0777:18786 315.0915:4243 341.0687:7119 353.0892:11221 359.0777:26256 371.2448:7903 429.7155:2117 489.1413:35332 490.1485:2926 533.1324:58318 533.2990:4203	0.0456	-0.35
11.8	534.5587		0.0001	0.61
14.4	535.3637		0.0316	-0.37
8.3	537.2201	50.9975:1691 55.0840:1716 56.0121:2062 59.0142:14817 60.3807:1643 68.4208:2136 69.8579:1844 71.0141:71684 72.9933:4049 73.0297:29867 78.9593:2892 78.9993:2033 83.0139:6088 85.0299:20349 87.0090:3702 89.0247:25255 95.0141:3696 96.9602:7379 96.9699:5022 97.0299:4726 99.0089:7604 101.0247:111761 113.0248:58314 119.0351:6516 122.1906:2111 125.0246:8037 126.9142:2273 128.9600:4601 131.0353:2823 143.0359:10944 149.0458:9426 150.0489:3544 159.0302:20298 161.0460:112907 162.0492:3696 169.7781:1994 176.9508:2928 178.3610:2406 179.0582:2101 191.0568:3090 203.0832:2899 227.1295:14467 263.0771:3587 269.5189:2580 294.9607:3698 304.7463:2178 325.0771:5594 327.0932:2611 327.1279:2348 329.1617:126421 330.1649:9660 358.1591:3622 375.0704:6696 444.1297:4093 445.1189:23119 489.1996:2712 490.1328:4005 490.2023:23977 491.2151:395989 492.2181:40767 537.0217:2592 537.2893:7639	0.0434	-0.35

Rt (min)	*m/z*	*MS/MS spectrum*	*p.value*	*rho*
9.3	537.256	54.6148:1647 59.0114:1601 59.0139:20155 70.8950:1753 71.0138:15553 73.0293:4380 77.6877:2109 80.0197:1734 83.0139:3041 85.0293:3285 89.0244:26330 101.0243:28808 103.0400:4530 112.5547:2152 113.0242:15150 119.0350:10055 125.0243:6884 131.0349:2844 135.0450:3436 136.0171:2163 143.0348:4801 149.0459:4006 151.0400:6340 159.0300:2935 160.3477:2123 161.0456:32604 163.0611:13561 165.0556:3684 177.0190:9403 179.0352:6629 179.0563:3461 191.0711:5395 205.0721:3473 221.0822:3204 257.0828:2205 259.0613:8893 287.0925:4794 297.1130:3783 311.5775:2287 313.0705:5012 326.1145:4959 327.1233:32224 328.1289:3031 329.0681:3391 329.1052:2818 329.1394:18606 330.1453:2576 339.0725:11946 339.1234:25441 342.1108:3827 345.1343:95906 345.1913:36847 346.1367:7742 357.0605:5737 357.1342:32223 358.1391:4798 360.1203:2625 375.1448:124213 375.2747:12269 376.1490:10376 447.1298:2222 488.8820:2265 491.2530:52268 492.2596:4717 537.2021:12133 537.3208:14839	0.0494	0.34
8.7	538.5747		0.0480	-0.34
11.2	545.26		0.0020	0.51
10.0	546.1613	51.0493:1520 52.7517:1526 59.0138:9464 61.9884:2108 66.6053:2238 66.9917:1980 71.0138:5386 72.9931:6174 78.9592:2998 87.0088:4289 89.0246:10850 96.9600:4984 96.9697:6713 97.5335:1781 101.0245:8064 109.0295:14190 113.0245:3654 116.9283:3139 119.035:5629 121.0292:2626 125.0241:3006 135.0457:2987 149.0457:3368 164.8185:2062 168.0826:5311 189.0920:2553 191.0565:3238 195.0671:2185 205.1226:3049 212.0715:12258 213.0806:3775 223.1343:21160 224.1374:2996 230.0828:8348 237.0617:4886 249.1143:4673 250.0884:4468 252.1016:3631 262.0874:48326 263.0941:2615 267.1243:22222 268.1284:5285 272.0940:3003 278.1185:23479 294.1142:4470 304.0985:17814 306.0777:21635 320.0944:6992 322.1086:21023 340.1191:43928 341.1221:3721 348.0880:31430 350.1045:3962 366.0987:108425 367.1020:4663 382.1313:5705 384.1094:70978 385.1144:4729 392.1390:11448 408.1085:16537 409.1058:2492 426.1202:20972 455.5861:2079 484.1569:2722 502.1708:12381 510.1418:13180 511.1474:3517 528.1516:110727 529.1567:9873 546.1630:130920	0.0316	-0.37
6.9	548.1209	60.4535:1980 75.9294:1870 76.4594:2258 88.6664:2097 123.0455:1724 180.9635:2474 203.4511:1912 282.5428:1894 315.9600:1928 329.2251:1977 336.0650:3563 435.1643:2158 456.1102:39201 457.0717:10989 458.1266:5797 491.0841:2135 501.0665:7195 502.1131:7925 503.1217:6039 548.1155:2830	0.0392	0.36
2.6	551.0168		0.0373	0.36
11.1	563.1782		0.0464	0.34
8.5	572.0105		0.0419	0.35

Rt (min)	*m/z*	*MS/MS spectrum*	*p.value*	*rho*
12.4	573.2557	59.0110:1908 59.0138:36324 59.5857:2004 65.9728:1733 68.0750:1910 70.9020:1600 70.9184:1750 71.0103:3002 71.0139:41497 73.0294:10764 83.0139:2918 85.0294:4228 89.0245:68703 90.9566:2069 91.5702:2249 97.0298:3117 99.0088:2250 99.0452:23793 101.0243:38356 103.0402:8025 113.0242:11021 115.0395:2470 119.0350:11568 125.0244:24009 129.0196:2346 131.0349:24391 143.035:7274 149.0457:24106 161.0454:4688 161.2476:1928 163.0614:19223 177.0201:2612 191.0563:10511 205.0719:18953 233.0653:3634 233.1548:83677 234.1556:2780 271.0601:2026 283.0251:16501 284.0321:4951 285.0404:38419 286.0439:3276 289.0726:2644 293.0879:16372 299.3877:2289 310.7101:2065 325.0358:7495 339.0509:4567 388.6770:2830 395.2426:2645 429.0650:3599 447.0716:26566 448.0779:6316	0.0313	-0.37
8.8	575.1914		0.0332	-0.37
10.0	579.0992		0.0376	0.36
11.3	591.1522	57.2665:1602 59.0138:3236 65.7636:2198 71.0139:2524 89.0245:6982 99.0453:3994 101.0244:2495 121.0297:2892 125.0244:20725 131.0347:4043 132.5926:2079 133.0147:2712 137.0247:15329 139.0403:5666 149.0248:8142 149.0450:3036 151.0407:5136 157.3619:2337 161.0250:4671 161.0460:4112 164.1342:2236 165.0189:2479 167.0352:5725 177.0194:5848 178.2960:2167 179.0354:4980 191.0339:3049 203.0727:2539 215.0711:36847 216.7530:2088 228.5074:2251 243.0297:4695 245.0823:15680 257.0814:13245 287.0569:3546 289.0720:204596 290.0761:8543 301.0396:5330 301.0719:151231 302.0755:4030 305.0672:3230 325.1314:2997 413.2193:3236 423.0761:2467 439.1036:25309 447.1003:2598 457.1721:7168 465.0853:2563 547.1612:2924 591.1504:5012	0.0424	0.35
2.3	592.0242	55.2722:1763 59.0319:1785 63.8747:2577 87.0087:3593 88.9886:2213 93.0773:4209 96.9602:4966 112.3763:2353 121.3370:2095 128.0354:9439 133.0135:5177 140.5991:2584 143.0457:4023 149.0091:161566 150.0128:4465 160.0074:2815 171.0779:3925 191.7406:2402 192.9292:2377 229.5422:2396 231.0000:4286 249.0592:6755 250.9446:8206 254.0799:4348 262.1689:2244 268.9538:12891 272.0899:10668 272.3111:2520 288.0656:5430 295.1051:3333 297.0491:2216 306.0765:91352 307.0815:4680 343.9672:4427 344.0317:101483 344.9653:3337 345.0347:7291 358.9502:4673 358.9926:3652 418.0284:2522 423.9918:9895 424.0440:2406 440.9421:3105 441.9942:12196 494.0416:35898 494.1033:7061 591.1793:8022 592.1843:3788	0.0240	0.39
12.1	602.1284		0.0456	0.35
9.3	606.1058		0.0484	0.34
12.4	623.0883		0.0420	0.35
2.0	636.2		0.0003	-0.58

Rt (min)	*m/z*	*MS/MS spectrum*	*p.value*	*rho*
2.2	642.0149	52.6680:1922 53.8182:1970 54.8065:1878 67.1449:2132 87.4305:1977 93.0754:2988 93.0800:2789 93.8906:2202 96.9695:1993 113.2106:2296 149.0088:14729 152.0908:2426 170.7322:2170 199.0012:2705 199.9408:4903 209.8918:2799 219.8817:2229 236.4844:2244 237.8973:43315 255.9081:4964 259.8572:2500 259.8859:2455 261.9421:11257 269.9138:3006 271.4919:2105 281.9017:2770 299.8979:4799 300.0498:5392 306.0782:2454 329.9426:8228 336.2695:2289 353.9438:6284 402.7137:2237 412.0308:66786 438.0498:5912 473.9864:7417 482.0356:15499 491.9958:11139 544.0371:50648 618.229:2831 640.7546:2911	0.0310	-0.37
1.8	642.7917		0.0407	-0.35
4.3	643.1162		0.0239	0.39
1.8	644.7897		0.0396	-0.35
16.2	651.246		0.0205	-0.40
10.7	658.2556		0.0211	0.39
12.8	659.2152	53.1793:1894 81.4792:2113 101.3662:2390 112.2642:2072 131.6938:2390 137.0240:2734 147.7351:2005 163.0401:2774 167.2815:2014 214.4214:2410 245.0823:5364 289.0715:46986 312.4778:2258 315.0875:38259 343.1188:10627 369.1345:39000 384.84:2379 399.0790:2869 418.0947:2317 507.1667:2882 615.9776:2279	0.0492	0.34
1.9	659.8798		0.0368	-0.36
9.2	667.0797		0.0456	-0.35
12.4	675.2093	69.5631:2627 96.1472:2299 116.9278:2107 125.0242:9589 130.7403:1989 137.0244:7226 139.0405:2254 147.5732:2147 161.0239:2852 163.0402:2331 165.4000:2162 167.0340:3785 177.0191:2852 179.0353:3276 189.7112:2500 205.0514:3238 231.0649:2482 245.0824:5131 279.1136:4003 289.0713:37643 290.0752:2945 305.0663:30312 315.0871:12135 323.1045:2999 331.0825:28082 343.1177:4829 359.1132:5800 369.1366:3721 385.13:28492 386.1357:3148 422.6630:2330 503.0936:3648 507.1617:2660 508.1628:2284 523.1642:3445	0.0029	0.49
1.8	677.3519		0.0334	-0.37
8.4	678.1857		0.0340	0.36
8.1	679.1858	68.4032:1891 78.7231:1995 99.5030:2084 101.8797:2299 114.6735:2220 115.0041:4713 135.0456:2695 137.0248:2191 163.0411:2666 173.0465:8271 179.0357:48502 180.0385:2576 183.5440:2346 187.5924:2289 191.0568:74134 226.0653:5390 227.0719:18572 255.1033:34575 256.1072:3342 299.0936:7337 332.9958:2418 336.0790:2322 346.0077:2841 389.1256:141683 390.1295:13645 487.1283:5292 505.1379:20536 578.2886:2679 587.0450:2517 679.1888:3254	0.0432	-0.35
9.1	681.2039	59.0139:418390 60.0171:3356 61.6076:2161 61.6158:2104 71.0138:9073 73.0292:4602 88.4802:2094 89.0248:2341 101.0245:10089 109.1911:2419 113.0242:4138 135.0457:2409 145.0296:81078 149.0090:4999 161.0457:24976 163.0402:22869 169.0147:3623 179.0353:5651 183.0244:2389 187.0406:5798 191.0564:3984 191.2408:2311 193.0504:12173 195.0658:2612 205.0506:2851 265.0707:3697 269.1031:131950 270.1064:8996 271.0992:3990 296.9693:3561 311.1152:10122 325.0928:24053 326.1036:2385 368.9563:6426	0.0340	0.36
10.2	685.0478		0.0033	-0.49
8.0	686.2747		0.0426	0.35

Rt (min)	*m/z*	*MS/MS spectrum*	*p.value*	*rho*
9.9	687.158		0.0410	0.35
2.0	688.9324		0.0066	0.46
9.1	690.0526	52.4001:1762 52.9549:2213 59.0139:29286 60.0173:2321 60.3158:1890 62.8286:2055 67.1184:1850 91.0553:2931 93.0731:3548 101.0246:2240 101.9160:2093 129.9368:3185 135.0446:2606 144.6083:2254 149.0093:7730 163.0402:3072 165.2729:2294 171.9467:4977 172.9551:2832 179.0351:7794 191.0571:3309 193.0506:7732 199.9415:17770 200.9487:2375 201.0168:9960 201.9208:4010 217.9672:15266 227.9367:2557 231.9834:4800 245.9628:7071 247.9786:14026 259.9782:3138 261.9421:4769 269.1036:6132 270.1061:2889 273.9585:3097 275.9729:54254 276.0013:3985 276.9796:5258 289.9516:5693 291.9698:7030 292.9746:8195 301.9736:84430 302.9769:3387 304.9741:3423 318.1743:4417 319.9580:58708 333.0240:6503 363.9725:6374 364.9603:23797 365.9636:2774 378.0057:2834 379.0111:5632 380.0193:4767 393.9990:9945 396.0150:27054 412.0126:3533 422.0314:5219 466.0216:13541 596.1830:2534 646.4998:2284	0.0113	0.43
7.5	691.2463	57.0443:2324 60.3506:1837 67.4048:2332 80.4658:1985 88.4147:2301 93.0808:3447 108.0228:7158 109.0299:3851 117.3602:2532 152.0116:11979 153.0193:14743 160.5779:2263 164.3499:2081 165.0194:2500 215.1288:2516 306.1938:2367 315.0732:370244 316.0760:32778 317.0781:4218 323.1833:3045 424.9636:4049 468.9534:7020 516.7261:2770 668.7394:2476	0.0376	-0.36
11.0	692.0613		0.0090	-0.44
7.9	703.2809		0.0417	0.35
4.7	709.1118		0.0385	0.36
9.1	711.2147	52.6788:2343 56.9192:1763 59.0140:14401 71.0136:4088 89.0244:11267 101.0245:5106 113.0242:4848 119.0350:8901 146.0307:2226 151.0386:2137 152.1421:2229 153.6221:2088 163.0402:16853 171.9464:49605 179.0353:4652 179.0559:3951 182.7867:2371 213.9575:17105 231.9675:11177 261.7045:3023 269.1021:3872 275.9737:6017 289.9889:2561 303.9676:7854 304.9762:6988 327.0970:6852 347.9918:4072 357.1349:16714 371.0880:5595 371.2062:5376 372.0931:7190 378.0050:9545 397.1059:4030 413.0949:16646 414.1029:2980 426.1051:7918 441.0914:9823 445.1213:7566 503.2498:32772 515.1289:47555 516.1301:4220 518.1302:3475 534.1363:2440 547.1545:10277 565.1669:4288 664.3698:3716 665.3005:19713 679.1798:4789 711.2021:6790	0.0021	0.51
8.3	717.2601		0.0089	0.44
2.1	719.0513		0.0430	0.35
10.0	727.2483		0.0150	0.41
8.8	736.0933	52.5969:1865 53.6311:1813 57.5219:2071 59.0139:4494 62.2124:2245 121.8440:2356 137.0244:2862 157.2574:2479 161.0248:30679 162.0288:3800 171.9472:4614 179.0356:24542 191.0572:4524 207.2638:2502 231.9682:3612 270.5375:2573 273.9564:3486 273.9832:3627 289.0727:6319 291.9669:27178 299.0572:3287 341.0885:30408 342.0924:8974 349.1873:2728 353.0901:5599 363.9924:2983 393.0438:3481 393.9998:15022 395.0092:8554 411.9881:11246 419.0792:9162 437.0906:7135 453.0338:4569 454.0215:273418 455.0260:31343 480.0367:10090 556.0496:28350 557.0537:5109 572.0386:2843 573.0529:4805 574.0428:128516 575.0481:15577 597.8446:2887 735.1037:6754 736.0970:554579	0.0474	0.34

Rt (min)	*m/z*	*MS/MS spectrum*	*p.value*	*rho*
9.3	749.0693		0.0147	-0.41
9.4	757.207	69.5408:2528 70.1527:2045 71.0136:3641 75.7544:2217 78.8253:2405 98.2414:2187 125.0238:2484 145.0294:4064 163.0402:6188 165.0198:2633 171.9464:22570 179.0343:3725 213.9571:6938 231.9675:5007 289.0720:3320 291.0716:2528 305.0664:4614 306.0707:2949 327.0968:15484 366.1212:11767 367.1243:2732 371.0848:10073 372.0932:5984 413.0967:22185 414.1022:6208 425.0942:3172 426.1051:10086 441.0921:3831 445.1216:4018 471.1406:3408 515.1281:146037 516.1291:6543 547.1541:15722 679.1731:7020 731.1234:3024	0.0335	0.37
11.7	767.0688	56.0014:1847 59.3435:1930 61.1630:1956 64.9109:2117 69.3595:2282 82.7186:2289 116.9283:6552 123.0079:4331 125.0243:7588 136.4151:2664 153.0186:4626 166.998:4570 167.0328:2411 169.0138:5218 177.0189:4904 229.0141:5054 247.0239:12070 248.0285:3179 253.9414:2345 257.0074:2543 270.9879:3281 273.0030:22144 274.0070:2919 275.0198:10336 286.9855:2629 298.9835:17721 300.9987:24173 301.0310:4281 302.0020:6588 339.0485:3122 341.0662:3029 427.0302:6164 443.0236:2779 445.0353:3310 465.1661:2227 499.4314:2696 581.0558:10932 597.0465:5666 656.2441:4221 657.2495:3919 705.0666:2986 721.0638:2733 749.0618:55293 750.0565:7187	0.0478	-0.34
7.5	768.1415		0.0428	0.35
10.4	772.1882	58.5792:1857 87.0089:8217 94.5132:2549 101.1538:2470 106.3339:2524 112.9882:3554 125.0245:5229 128.1599:2276 141.9360:2797 149.0093:5416 156.9593:3129 163.0402:28972 169.9304:2968 171.9467:5250 177.0199:11058 178.4660:2212 179.0354:9768 186.9708:4923 193.0512:10367 199.9412:4844 200.9495:5928 201.9564:2505 213.9574:23757 214.9649:7186 229.9525:7899 230.9600:3115 263.1161:2729 268.7997:2743 271.9649:3674 276.9784:2637 295.0446:3346 301.0348:3099 303.0516:2697 305.0678:8190 306.0665:2627 376.1213:4766 389.1271:15099 418.1302:14262 432.1108:27445 446.1247:3589 447.1320:3217 474.1206:4613 476.1345:20002 494.1476:24093 495.1508:6414 520.1639:13526 536.1532:6769 564.1524:204742 565.1552:14424 567.2419:4975 578.4869:2864 581.2340:2808 632.3334:2639 639.2615:6623 640.2670:3313	0.0082	0.45
2.0	792.1841		0.0282	-0.38
1.9	794.9412		0.0190	-0.40
10.4	798.557		0.0428	-0.35
1.9	800.6951		0.0262	0.38
7.8	801.3508		0.0477	0.34
1.9	812.713		0.0156	0.41
9.5	816.2376	59.0117:4171 59.0141:3961 65.2554:2108 87.0086:3580 89.0245:5473 92.9114:2274 101.0247:4727 113.0251:2994 113.0820:2775 118.7339:2476 119.0348:3717 125.9268:2763 127.0967:2294 142.0570:2819 142.0663:15075 149.0091:4212 160.0619:4337 169.0309:2705 179.0342:4411 186.0562:112337 186.0731:5628 187.0599:5795 189.7826:2589 196.0469:2444 204.0666:58495 205.0698:3417 214.3648:2367 225.4484:2811 226.4913:2736 246.0769:12040 273.0048:6402 275.0207:4554 287.0564:5803 305.0672:5755 366.1196:881703 367.0798:4621 367.1233:59878 404.0675:5053 449.1087:4328 465.1773:3097 479.0842:4257 543.5653:2673 567.2684:2631 613.0439:8646 620.3096:3342 767.4269:2878 771.1893:4332	0.0498	-0.34

Rt (min)	*m/z*	*MS/MS spectrum*	*p.value*	*rho*
11.8	817.678		0.0123	0.42
1.8	818.3272		0.0202	-0.40
11.4	819.2346		0.0212	-0.39
11.3	825.1733		0.0497	-0.34
1.9	830.8974		0.0426	-0.35
10.9	840.6289		0.0132	-0.42
14.2	845.4908	67.1532:2130 68.9525:2203 91.8895:2299 98.5289:2393 98.7635:2546 107.6021:2569 112.9853:4464 118.4387:2127 125.2155:2383 171.1026:17236 235.0819:3140 235.6280:3063 253.0925:8939 253.1448:5011 271.1557:3086 277.2171:68512 278.2205:6995 303.1807:51730 367.5850:2417 581.6975:2677	0.0147	0.41
10.8	846.5042		0.0366	-0.36
2.1	852.9855		0.0096	0.44
1.7	861.7747		0.0405	0.35
10.8	864.5158	61.2074:2503 69.0689:2265 69.8256:2150 91.0800:2487 125.0242:108343 135.2082:2624 137.0241:5651 151.0395:3706 161.0239:27299 161.0334:2205 163.0042:4707 165.0198:4719 169.7894:2911 175.0417:2827 177.0194:8821 203.0348:3732 209.0065:2922 212.4435:2540 214.9994:8185 216.0031:3923 217.0147:4173 218.0209:2750 219.0297:7245 221.0106:2780 221.0451:5111 229.0142:2674 231.0306:2957 233.0103:3305 235.024:3668 236.2478:2713 242.9923:8368 243.0296:29624 243.0677:3154 243.9984:2734 245.0098:15215 245.0452:20919 245.0821:4694 246.0131:9598 247.0251:46796 248.0282:31017 249.0315:12927 250.0332:2740 251.0438:4041 255.0313:2839 258.9877:3732 259.9949:6274 261.0027:16535 261.0391:19965 262.0073:9505 263.0177:6679 264.0201:3683 270.9891:9612 271.9917:4418 273.0043:46752 273.0354:5163 274.0078:32856 275.0162:30532 276.0219:13108 277.0249:11562 285.0424:4475 286.9850:16662 287.0558:62627 287.9865:9234 288.9962:7724 289.0715:80715 290.0032:9520 291.0151:28772 292.0182:19476 293.0190:11134 294.0233:7819 298.9833:68792 299.0553:10243 299.9876:49695 300.9985:119894 302.0021:72513 303.0041:47995 303.1974:2939 304.0075:22297 315.0881:2991 316.9922:5510 319.0004:3847 329.0684:3846 337.0714:3887 391.0494:3421 405.0611:21868 406.0499:3429 407.0771:73198 413.0864:7143 423.0691:6576 425.0872:17688 435.0609:10626 436.0598:7495 437.0652:6627 438.0684:3276 449.0349:7663 449.0889:13466 450.0368:7448 451.0458:4810 451.1022:14076 452.0450:4042 517.1150:4013 525.0823:4273 543.0882:5699 559.1221:3221 561.1059:4059 575.1160:4777 577.1353:9839 627.2051:3915 682.0681:4342 695.1350:7856 710.0590:9088 728.0698:7444 734.0325:5971 735.0350:6248 736.0417:33359 737.0322:4078 738.0519:9772 752.0522:5220 753.0461:6602 754.0485:38727 755.0524:5394 756.0621:5864 800.6478:3361 843.4071:3233	0.0235	-0.39
8.9	867.2961		0.0256	0.38

Rt (min)	*m/z*	*MS/MS spectrum*	*p.value*	*rho*
7.9	870.1163	62.5579:2356 75.4181:2017 78.9589:30111 85.6954:2368 96.9598:152574 96.9694:122726 102.8135:2296 109.4505:2513 137.0242:4610 163.5709:2651 179.0346:2878 213.9567:5386 259.0233:5965 273.9776:32406 285.0410:4360 301.9723:37875 315.9884:6751 327.9958:3136 344.9127:2910 376.0095:142966 377.0126:8080 397.9752:5716 447.0943:10466 500.0043:12412 501.0120:15829 518.0175:2713 542.0167:7775 560.0257:16236 588.0193:3662 602.0386:8456 662.0574:126020 663.0584:14133 680.0634:4190 824.1147:39751 824.2692:5373	0.0306	0.37
9.8	875.3016	61.0167:2236 70.8852:2275 75.2365:2525 93.0812:4920 100.2058:2757 135.0456:4502 140.7482:2288 149.0093:77761 150.0126:4975 157.1695:2615 161.0245:10230 161.2493:2634 179.0353:73728 180.0385:10846 191.0564:786172 192.0600:17201 198.6389:2604 209.8768:2431 271.6504:2563 311.0430:5853 353.0887:73037 354.0915:5905 362.4287:2645 416.0269:2726 435.2650:2699 516.3996:2758 732.5495:3556	0.0381	-0.36
9.3	891.5859		0.0397	-0.35
12.5	892.1837		0.0065	0.46
10.2	895.1742		0.0338	-0.36
12.2	901.2028	70.4858:2386 84.1869:2673 100.0327:2721 104.2446:2487 107.4629:2419 123.2721:2378 125.024:13102 128.3498:2555 161.0241:6317 177.0191:30111 206.2803:2603 206.4077:3181 238.103:2365 249.5188:2723 284.1262:2830 287.0564:16669 289.0716:5698 299.0556:3779 321.0372:2874 339.0516:15201 341.0663:43083 449.0887:2721 451.1032:19531 452.1044:4624 462.5080:3367 503.0963:3087 569.1063:8661 595.1246:6066 613.1284:3032 639.1137:4885	0.0279	0.38
10.0	907.2402		0.0054	-0.47
10.2	924.1514		0.0195	-0.40
1.9	926.6338		0.0304	-0.37
9.3	927.0514	128.0346:1504 133.7424:1428 143.0456:1695 169.0137:2085 177.0558:2059 217.0116:3053 225.0761:1950 245.0095:1749 247.0238:4202 249.0394:2437 261.0036:6685 272.0883:4294 273.0037:6342 275.0195:3625 291.0140:5486 298.9825:10458 300.9986:7158 306.0752:5976 373.1502:2234 387.0367:4170 401.0142:2905 421.5227:2303 431.0248:4049 449.0366:21184 450.0409:2736 461.0364:5357 463.0478:4747 479.0471:5377 601.0511:3018 676.9951:4960 688.2737:3545	0.0050	-0.47
12.3	943.4579	65.7874:2536 80.8347:2310 83.9798:2808 87.0084:2792 87.4653:2692 101.8506:2719 115.0036:16873 125.0244:3902 166.0407:2801 175.0246:72944 253.0935:3096 309.2066:7357 357.8269:2458 411.3019:2921 422.2526:2801 554.9925:3013 700.4818:3460 761.4180:2725 825.4262:4100	0.0401	0.35
11.3	950.3258		0.0332	0.37
10.2	963.5922		0.0328	-0.37
9.4	979.2382		0.0133	0.42
1.9	988.8901		0.0173	0.41

Table S7 Features in positive ionization mode [M+H]+ correlated with Fv/Fm (P < 0.05). Features are reported with their m/z, retention time (Rt min) and MS/MS spectrum when collected. Spearman correlations were calculated (rho).

Rt (min)	*m/z*	*MS/MS Spectrum*	*p.value*	*rho*
7.6	126.9722		0.0111	-0.43
9.1	131.0494	84.0455:1245 103.0547:10514 131.0490:2686	0.0227	-0.39
7.6	141.9588	56.9432:8517 67.0550:1259 70.6532:1145 72.9375:1186 73.0553:1158 82.0144:3533 83.0920:1168 93.2759:1174 97.9694:8651 99.5645:1172 100.0248:2461 113.9642:9397 117.0057:1324 118.9433:1438 139.6774:1363	0.0064	-0.46
6.5	144.0212		0.0225	-0.39
4.1	147.0438	52.7935:1233 59.5310:1023 59.7815:1035 61.3428:1089 68.8194:1007 72.6394:1238 78.7125:1145 80.0221:1272 82.0142:1747 84.0450:5044 91.0548:2166 100.0248:1492 108.5920:1191 110.0090:2225 111.0095:1699 121.2791:1203 128.0190:1589 130.0498:1780 147.0439:3633	0.0283	-0.38
1.7	148.0186		0.0490	-0.34
7.8	175.1485	53.3795:994 61.9790:1020 78.8540:1193 81.5672:1100 97.0292:3009 98.9860:1105 114.0559:1251 119.0865:2718 133.1015:2991 147.1715:1313 174.0765:3432 175.0777:2186 175.1192:1589 175.1478:6367	0.0250	0.38
4.1	185.0446		0.0472	-0.34
7.8	193.1593	53.8721:1246 58.0659:1808 62.4890:1203 63.3692:1077 65.0562:1205 65.1093:1147 70.7460:1131 72.1379:1295 85.0653:2666 87.0324:1316 99.0809:2810 101.8535:1378 105.0703:2096 107.0865:1557 107.9605:11283 109.1012:1928 119.0862:1765 133.0648:3157 133.1011:5342 135.0440:4940 135.1168:5495 137.0963:3768 151.9498:4088 161.0594:2463 175.1481:3977 183.6658:1370 193.1588:4418	0.0341	0.36
2.6	195.1230		0.0240	-0.39
8.1	199.0602	53.6027:1059 64.5234:1369 77.0394:1346 80.3948:1342 81.0584:1358 95.0497:7443 105.0452:2319 106.9921:5394 116.6802:1190 121.0852:1308 121.1012:2233 123.0442:7572 125.0236:1711 131.5441:1322 140.0466:42590 153.0543:1602 155.0704:10994 155.0825:2386 156.0871:3305 158.0026:3836 181.0490:2711 199.0608:2139 199.0997:1492	0.0371	-0.36
3.6	216.0325		0.0400	-0.35
1.9	224.0275		0.0246	0.38
9.8	229.9967		0.0276	-0.38
9.1	239.0917		0.0316	0.37
11.1	249.1116	82.5166:1469 90.7668:1738 159.0810:3235 177.6103:1324 187.0755:4102 204.0782:2211 221.1175:2464 249.1119:20701	0.0164	0.41
7.6	249.9800		0.0391	-0.36
4.1	253.1283		0.0264	-0.38
11.0	253.2175		0.0333	0.37
12.9	255.1021		0.0197	0.40
9.1	257.1021	51.7467:1208 53.4843:1373 61.0293:7497 63.9568:1264 66.5159:1416 81.0705:3823 83.0862:1514 90.0556:1937 91.0396:6750 93.0706:2343 107.0498:1797 109.0657:2358 111.0810:18772 123.0806:2584 137.0596:3349 139.0752:2039 151.0754:2072 155.0703:2234 165.0919:1614 167.0694:1909 179.0437:1714 195.1016:5476 211.0966:8363 239.0919:1904	0.0481	0.34
10.8	263.0187		0.0196	-0.40

Rt (min)	*m/z*	*MS/MS Spectrum*	*p.value*	*rho*
5.1	264.1444	61.0295:1607 66.1359:1222 69.0343:2354 69.0706:1538 76.2500:1260 85.0291:40480 97.0288:5763 102.0919:60642 102.0986:3352 109.0288:4039 125.1577:1440 127.0395:7712 138.4827:1345 145.0497:4118 145.8851:1587 187.276:1849	0.0250	0.38
2.3	272.0884	52.8918:1292 53.2559:1129 56.9660:1266 61.0291:1753 61.8191:1514 67.6058:1488 68.6515:1392 69.0344:4302 81.0343:1952 81.2178:1355 85.0292:22141 90.0558:1671 91.0396:1717 92.0502:4237 97.0291:4872 98.9851:3766 99.0448:4917 103.0401:2621 109.0290:2051 110.0607:93807 111.0643:2030 115.7697:1442 122.0605:2913 127.0396:6273 134.0604:4271 139.0392:1825 145.0497:3766 152.0708:4160 155.3865:1460 158.0568:4016 168.0772:2068 181.2501:1511 199.0088:1536 212.0662:3356 226.3393:1501 254.0790:1872 270.2475:1456 272.0879:4670	0.0263	-0.38
13.6	284.0718	60.0815:2147 70.0406:6154 83.2218:1310 83.9080:1599 158.9764:37251 172.9920:11428 284.0722:4093 284.1684:4820 284.3312:369803	0.0274	-0.38
13.6	289.2528	69.0704:1525 77.7035:1402 78.1439:1257 81.0700:3217 93.2611:1458 95.0857:2888 105.0701:1862 106.5679:1284 107.0851:2217 109.1009:4193 121.1006:2951 127.1113:3482 135.1164:3076 147.1158:2350 149.1316:6458 159.1681:1537 163.1471:2648 187.1483:2826 193.9065:1656 201.1620:1717 203.1789:7705 239.6633:1792 261.9581:1548 270.177:1602 271.2413:3332 281.0708:1605 289.2515:1645	0.0391	0.36
14.3	289.2535	50.2698:1200 52.2141:1295 55.5853:1183 62.2772:1522 81.0706:1714 82.3238:1447 90.2514:1518 95.0864:2151 105.0705:1839 107.0864:3086 109.1019:4365 120.2694:1377 121.1015:3979 127.1122:2633 132.5359:1627 133.1012:2579 135.1176:2331 147.1172:3151 149.1326:5068 173.1328:1803 201.9996:1568 203.1797:6488 271.2402:2410 289.2521:2156	0.0328	0.37
2.2	290.1347	58.0659:117815 64.8641:1265 68.0502:1406 72.0556:1605 76.3002:1373 81.0341:2167 84.0449:7068 88.6800:1242 93.0614:1450 94.5712:1400 94.7144:1372 97.0292:2290 98.9846:28976 110.8867:1516 114.0552:11150 120.5145:1522 127.0390:4134 127.0497:1964 129.0550:1985 129.5916:1599 130.0499:7484 138.9645:1880 161.0920:41717 169.0978:2198 169.4423:1402 170.0809:2725 180.9744:7001 181.976:9311 191.0386:3149 196.9693:5249 197.0925:1903 197.9704:4820 198.9859:4954 199.9855:8997 200.0444:4492 200.9832:2193 209.9706:1590 214.9801:4725 215.1025:16333 215.9819:5421 216.9784:2023 227.1028:5781 227.9802:1675 228.9798:2012 244.1183:1979 250.3812:1554 272.1137:4942 273.1099:3578 276.0834:1640 289.9823:2165 290.0920:3216 290.1237:12606	0.0259	-0.38
15.8	293.2481	52.9210:1114 57.2355:1289 62.8728:1295 65.5931:1510 67.0551:12486 69.0709:1438 81.0706:13869 93.7519:1358 95.0861:14737 109.1017:10951 123.1175:8236 126.6651:1583 135.1169:2430 137.1329:3936 142.4329:1592 293.2478:3035	0.0163	0.41
5.1	302.1003	67.5394:1268 70.6839:1611 73.7911:1369 75.8938:1456 78.9579:1545 85.0292:7572 95.0497:2596 97.0290:6832 112.0759:3182 112.1507:1430 127.0380:1519 139.7663:1576 140.0705:29711 154.0867:1588 159.8292:1688 182.0815:15031 204.2731:1586 206.0814:4413 211.7246:1609 248.0917:2512 284.1677:3280	0.0106	0.43
7.7	307.0109		0.0349	-0.36
8.2	321.1110		0.0265	-0.38
2.3	346.0476	51.7172:1263 56.0503:1791 65.1274:1527 70.0660:3545 70.9025:1295 75.1480:1558 89.6207:1445 102.0258:1495 102.0514:1444 113.1375:1595 121.0350:1764 130.3747:1707 136.0619:4045 162.1553:1648 214.0828:6293 219.5254:1690 224.6608:1616	0.0301	0.37
8.9	346.2336		0.0354	-0.36

Rt (min)	*m/z*	*MS/MS Spectrum*	*p.value*	*rho*
11.4	350.1627	57.6599:1370 64.1013:1588 69.0338:1448 91.0551:2920 105.0702:2333 133.1014:14852 145.1015:5925 163.1120:26641 171.0323:19573 175.5579:1607 200.0597:4774 229.7551:1779 234.6215:1720 330.3182:1796 332.1520:8054 350.1628:7873	0.0438	-0.35
8.4	355.1733	64.5195:1407 67.4456:1270 69.0731:1392 94.2670:1644 95.3035:1551 98.5170:1600 128.6274:1760 163.0386:2456 191.7040:1542 354.0801:2118 355.1727:90563	0.0303	-0.37
7.6	365.1058		0.0214	-0.39
7.7	367.0322		0.0099	-0.44
7.6	371.0060		0.0206	-0.40
2.6	374.2018	53.8758:1345 57.2022:1550 85.0294:1909 89.0603:23849 130.0650:7913 132.0813:2706 133.0860:16663 139.4637:1861 141.9498:1893 151.0963:3233 176.0702:8941 177.1113:4221 178.0858:2338 194.0810:8638 195.1226:71326 196.1259:2739 256.9689:1947 330.5543:1710 374.1450:2910	0.0122	-0.42
2.6	379.1587		0.0415	-0.35
2.6	402.2343		0.0389	-0.36
7.6	406.1645	52.8327:1328 54.8549:1345 56.8772:1646 57.0344:2175 58.2442:1551 59.7167:1405 61.4943:1461 76.0224:7052 81.0708:3639 84.0450:2084 87.0270:1565 98.0971:6245 99.0811:7011 100.2361:1583 102.0205:1640 102.0557:2818 103.0831:1737 104.2279:1660 116.0168:3004 126.0874:2203 129.0658:2807 130.0503:6559 130.0679:2192 131.1322:2063 144.0113:3012 156.0846:15407 156.1021:6936 157.0680:10279 162.0221:28130 170.4245:1895 174.0951:19703 179.0482:11639 192.4253:2040 210.1121:3082 216.0692:12034 226.0369:1731 233.0596:2887 259.1116:101821 260.1162:3215 285.4704:2334 313.1237:3271 331.1333:8075	0.0307	0.37
8.4	409.1840	57.1930:1421 63.0956:1576 409.1830:181083	0.0018	-0.52
7.6	412.0324		0.0259	-0.38
9.8	415.9936		0.0424	-0.35
7.7	424.2547		0.0371	0.36
10.3	434.2759		0.0386	0.36
7.7	457.0796		0.0234	-0.39
10.6	462.1209	50.5375:1525 50.8761:1872 52.0095:1785 70.0382:2926 70.0420:48301 85.0305:7943 95.8118:1974 97.0589:2151 109.0307:6812 127.0417:13680 132.6378:1859 145.0522:3387 152.9196:1805 158.9794:4538 213.0261:9481 235.0305:2975 282.0616:19079 300.0718:116490 301.0743:7313 337.0247:20971 362.3212:2052 462.0823:2094	0.0400	-0.35
10.6	464.1185		0.0072	-0.45
9.1	490.1957		0.0415	-0.35
8.0	520.3337	53.9191:1510 61.3025:1795 83.0169:1513 83.3884:1742 87.0447:3965 89.0604:53687 89.8828:1793 93.0581:4028 95.8782:1837 96.4140:1683 97.6671:1742 132.7560:1915 133.0862:40904 154.7889:1819 170.8215:1868 177.1121:9023 188.0706:3057 202.4809:2076 229.0274:2170 485.0189:1939 501.2797:2208	0.0150	-0.41
9.0	528.1358		0.0429	0.35
11.8	544.3644	51.5988:1700 53.3846:1718 59.2273:1653 63.3100:2240 69.0706:2062 81.5127:1990 97.0655:9252 98.0971:15274 99.0808:7778 142.1228:300324 143.1267:9500 161.4345:2023 215.1083:2446 243.1020:5502 261.1132:16456 544.3586:8779	0.0308	-0.37
14.9	545.3846		0.0268	0.38
2.2	547.0610		0.0403	-0.35

Rt (min)	*m/z*	*MS/MS Spectrum*	*p.value*	*rho*
1.9	553.0831		0.0389	0.36
9.1	555.3361		0.0081	0.45
1.9	564.1487		0.0363	0.36
8.1	564.3585		0.0418	-0.35
11.0	577.1370	52.7772:1786 56.4824:1659 61.3650:1802 63.2072:1674 78.8399:1745 120.2671:2254 123.0448:19424 127.0397:10826 133.0656:1983 135.045:13612 139.0396:17166 151.0395:7587 163.0397:21810 163.3870:2095 237.1502:2219 245.0445:4388 247.0610:13325 257.0434:2380 261.0750:3324 271.0616:11375 287.0563:14748 289.0719:5167 379.0814:4110 407.0769:6111 409.0926:2793	0.0496	0.34
8.2	608.3858		0.0354	-0.36
14.4	691.4442		0.0336	0.37
12.1	746.4850	69.0706:5019 73.9870:2127 75.0448:5792 86.5482:2579 87.8971:1918 97.0655:11737 98.0970:24737 99.0447:10464 99.0810:9060 106.7913:2662 117.4006:2305 125.0599:2995 142.1228:427386 143.1265:15260 157.0859:6134 160.1332:4183 189.1121:12619 230.3523:2536 343.021:2599 451.0901:2785 460.0214:2760 669.8497:2865	0.0164	-0.41
14.7	755.512		0.0477	0.34
9.5	767.4202		0.0302	0.37
8.5	773.2139	55.5012:2310 56.0583:2187 67.3434:2071 69.0341:4886 69.3144:1967 69.5338:2320 75.3626:1957 77.9202:2354 80.1046:2049 85.0289:25618 91.0394:2965 97.0290:5838 122.0668:2372 127.0389:10532 133.0441:2844 136.4999:2475 145.0490:8390 168.0364:2633 178.0724:2674 207.1383:3372 217.4298:2475 274.5759:2266 287.0546:339860 288.0583:21328 329.0641:3245 449.1074:108494 450.1102:10092 611.1606:3911	0.0054	0.47
16.4	782.5699		0.0418	0.35
9.5	858.3699		0.0420	0.35
1.9	873.2644		0.0425	-0.35
